# Bridging Time-series Image Phenotyping and Functional–Structural Plant Modeling to Predict Adventitious Root System Architecture

**DOI:** 10.34133/plantphenomics.0127

**Published:** 2023-12-21

**Authors:** Sriram Parasurama, Darshi Banan, Kyungdahm Yun, Sharon Doty, Soo-Hyung Kim

**Affiliations:** ^1^ School of Environmental and Forest Sciences, University of Washington, Seattle, USA.; ^2^ Department of Smart Farm, Jeonbuk National University, Jeonju, Korea.; ^3^ School of Integrative Plant Science, Cornell University, Ithaca, NY 14853, USA.

## Abstract

Root system architecture (RSA) is an important measure of how plants navigate and interact with the soil environment. However, current methods in studying RSA must make tradeoffs between precision of data and proximity to natural conditions, with root growth in germination papers providing accessibility and high data resolution. Functional–structural plant models (FSPMs) can overcome this tradeoff, though parameterization and evaluation of FSPMs are traditionally based in manual measurements and visual comparison. Here, we applied a germination paper system to study the adventitious RSA and root phenology of *Populus trichocarpa* stem cuttings using time-series image-based phenotyping augmented by FSPM. We found a significant correlation between timing of root initiation and thermal time at cutting collection (*P* value = 0.0061, *R*^2^ = 0.875), but little correlation with RSA. We also present a use of RhizoVision [1] for automatically extracting FSPM parameters from time series images and evaluating FSPM simulations. A high accuracy of the parameterization was achieved in predicting 2D growth with a sensitivity rate of 83.5%. This accuracy was lost when predicting 3D growth with sensitivity rates of 38.5% to 48.7%, while overall accuracy varied with phenotyping methods. Despite this loss in accuracy, the new method is amenable to high throughput FSPM parameterization and bridges the gap between advances in time-series phenotyping and FSPMs.

## Graphical abstract

**Figure:**
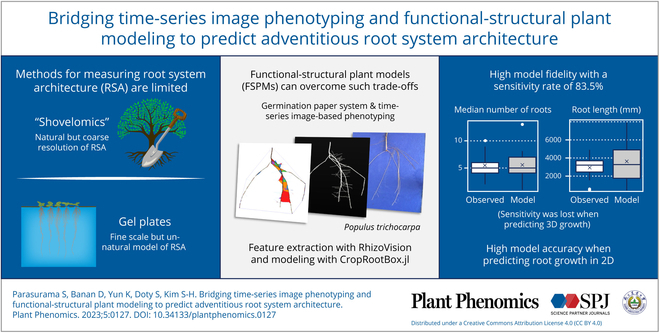

## Introduction

The root system of a plant is its navigation and anchoring mechanism in the belowground environment and is responsible for water, nutrient, and carbon flow in the rhizosphere [2,3], or the soil–root interface. Root system architecture (RSA) is the 3-dimensional (3D) structure of plant roots and is important for sink and storage functions, water and nutrient uptake, exchange of biochemical compounds, and association with symbiotic microorganisms [3–6]. Because of the uneven distribution of water and nutrients across the soil, plant survival depends on the timing and location of root initiation [2,3,7].

However, RSA is greatly understudied due to barriers such as the opacity of soil and fragility of finer roots [3,5,8]. Current methods exist on a tradeoff between accuracy of data and proximity to natural conditions. Destructive measurements such as “shovelomics” [9] and root washing may represent realistic root structures but offer very coarse data such as rough biomass or root length and result in a loss of information about spatial distribution of roots. Small-scale measurements on gel plates [10] on the other hand, may offer finer data but sacrifice their proximity to reality. Rhizotrons (or rhizoboxes) and x-ray computer tomography are study systems that exist in the middle of this tradeoff, balancing finer data and greater proximity to reality [3,8,11,12]. However, while x-ray computer tomography scanning [3] can accurately capture RSA, it can be expensive, time-consuming, and inaccessible to many researchers. Soil rhizoboxes, while cheaper and more accessible, can still be time-consuming to assemble and difficult to build for a large sample size [11]. A phenotyping method similar to rhizoboxes that uses germination paper can be a more effective and high-throughput alternative [3,8,13].

Ultimately, however, these methods are all limited in some way, having to exist along the tradeoffs between accuracy of data and proximity to natural conditions. Computer modeling can be a powerful approach to break past these restrictions. Specifically, functional–structural plant models (FSPMs) simulate plant architectural development alongside process-based functions, recognizing that both structure and function are necessary for a plant to grow [14,15]. CropRootBox.jl (written in Julia) is an FSPM simplified from CRootBox (written in C++) that can accurately simulate RSA development from user-defined parameters [16–18]. Historically, the parameters for these models were generated through manual measurements or “visual comparison” from literature image sets [16,19,20]. In recent years, developments in open-source root image analysis software such as RhizoVision allows for high-throughput feature extraction and, when coupled with a system such as germination papers, can lead to more accurate and efficient parameterization methods [1,13].

Despite these advances, most root phenotyping and modeling are tailored toward seed-based model organisms, while woody perennials and other vegetatively propagated plants [21] are largely ignored despite their importance in biological, ecological, and production contexts [8]. For instance, vegetatively propagated trees such as *Populus trichocarpa*, or black cottonwood, play a crucial role as early colonizers of disturbed ecosystems, in industrial pulp and biofuel production, and as scientific model organisms [22–25].

In production or ecological settings, stem cuttings are established through their adventitious roots, or those formed by nonroot tissue [26]. *P. trichocarpa* is often found along regularly disturbed flood banks, where broken branches that are fully or partially submerged form adventitious roots [27,28]. Similarly, nurseries propagate *P. trichocarpa* by burying stakes to promote root formation [28]. Studying the adventitious RSA [29] of *P. trichocarpa* could inform us of how and why it is superior in asexual propagation while many other *Populus* species and hybrids are not [25].

Nurseries must consider environmental and developmental factors such as seasonal timing when harvesting vegetative cuttings [30]. While phenology and its mechanistic basis have been well characterized in aboveground features of *Populus* species, fewer studies have examined seasonal relation with RSA in vegetatively propagated trees [31–35]. However, variation in *Populus* root biomass has been observed in response to shifts in thermal time [34] and rooting ability has been correlated with aboveground developmental events such as bud burst [35], indicating a potential relationship between RSA and timing of cutting harvest. Quantitatively defining this interaction from root initiation to full root system development is important to guide nursery cutting harvest, especially in a shifting climate.

Here, we aim to meet the following objectives:

1. Adapt a germination paper system [13] for vegetatively propagated cuttings.
2. Apply this system to study the effects of the timing of stem cuttings on adventitious RSA of vegetatively propagated *P. trichocarpa.*
3. Use features automatically extracted from root images to parameterize and evaluate a simple FSPM [14].

This process is designed to be affordable, high-throughput, and accessible.

## Materials and Methods

### Experimental design

**Fig. 1.**
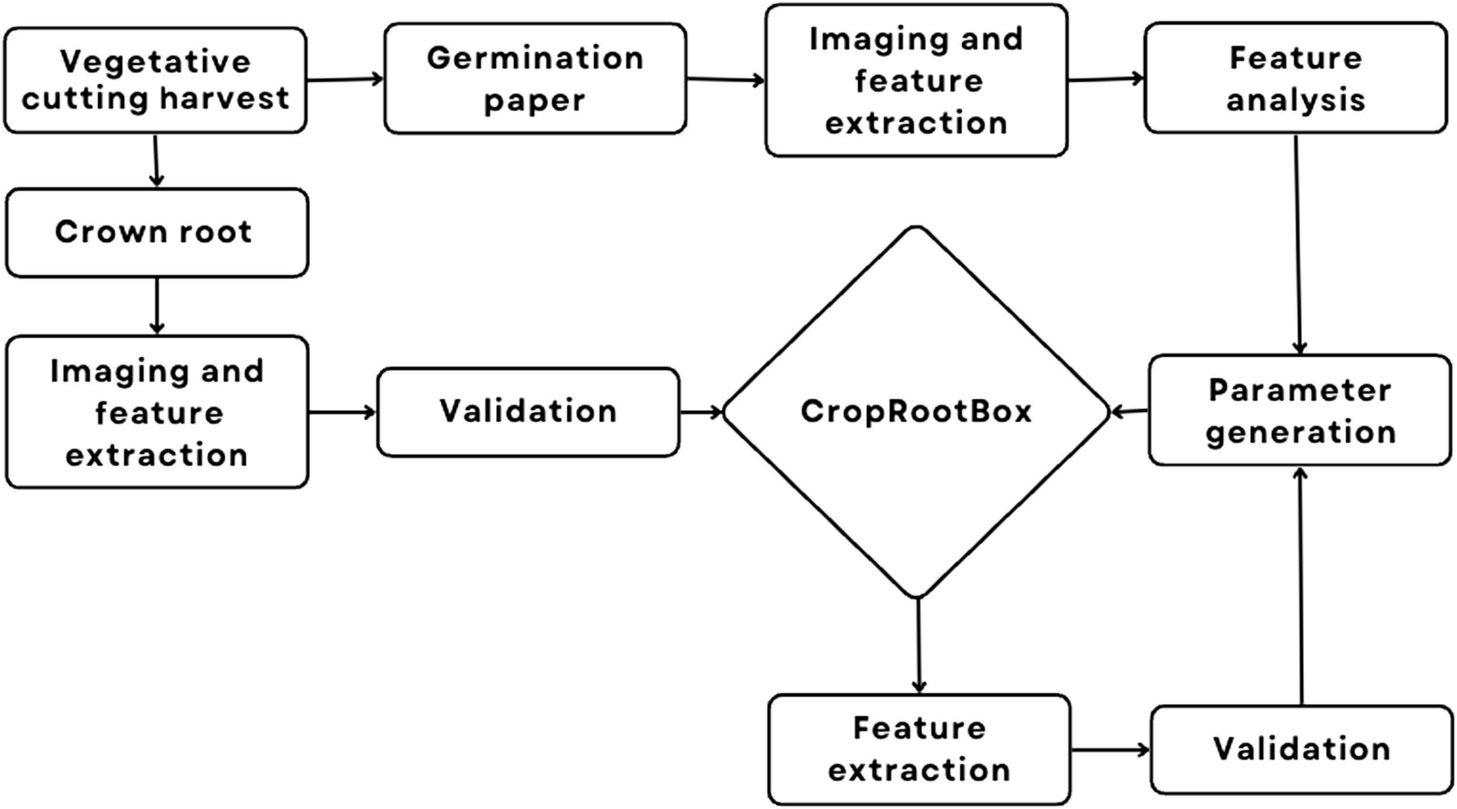
Flowchart diagram of the workflow presented in this phenotyping and modeling platform. The workflow begins with the vegetative cutting harvest and ends with CropRootBox.jl.

The study system uses *Populus trichocarpa* as a model organism to investigate the adventitious RSA in response to seasonal variation. This phenotyping and modeling platform has 11 interacting components, presented in Fig. 1. This process starts with harvesting the vegetative cuttings that are then rooted and transferred to germination paper. They are then imaged weekly and features are extracted for model parameter generation. From the model output, features are re-extracted to validate the parameter generation. A subset of cuttings are also harvested to be grown in Deepots to extract their crown roots and validate the model.

### Propagation and thermal time

Stem cuttings were collected from *P. trichocarpa* (Torr. and Gray) “Nisqually-1” clones maintained as coppices at the Center for Urban Horticulture, Seattle, WA (47.657, -122.290) 6 times monthly between 2022 October 15 and 2023 March 15th. The start date was around bud set and the end date was around bud burst of the coppices. At each collection date, 30 to 40 woody apical cuttings measuring 150 to 180 mm in length with 3 to 5 leaf nodes were harvested evenly from a random subset of trees. The cuttings were placed in darkened beakers filled with tap water, without any nutrient solution or rooting hormone, and kept at an average temperature of 20 °C in a greenhouse. Adventitious root initiation, defined as the first observation of a stem-borne root greater than 3 mm, was tracked daily. The time in days until 50% of the population had adventitious root emergence (T50) within each collection cohort was estimated using drc, an R extension package [36]. A subsample centered around the median time in days to adventitious root initiation with similar root lengths was then transferred to germination papers to evaluate their RSA.

Daily outdoor temperature data was tracked on the Seattle station of Washington State University’s AgWeatherNet [37]. This temperature data was used to calculate different measures of thermal time. Chilling units (CU; unitless), a measure of cold time, were calculated following equations from Hänninen [38] and parameter values from McKown et al. [33] and then summed to calculate cumulative units (Eq. 1).

$$\mathrm{CU}=\left\{\begin{array}{c} \;0,\;\text{if}\;T\left(t\right)\;\leq -3.4{}^{\circ}C\;\text{or}\;T\left(t\right)\;>10.4{}^{\circ}C\\ 0.159*\;T\left(t\right)+0.506,\;\text{if}\;-\;3.4{}^{\circ}C<\;T\left(t\right)\;\leq \;3.5{}^{\circ}C\\ -0159*\;T\left(t\right)\;+\;1.621,\;\text{if}\;3.5^{{}^{\circ}}C<\;T\left(t\right)\;\leq \;10.4{}^{\circ}C \end{array}\right.$$ (1)

Forcing units (FU; unitless), a measure of warm time, were similarly calculated following equations and parameters from Harrington et al. [39] (Eq. 2).

$$\mathrm{FU}=\frac{1}{e^{\left(-0.47*T\left(t\right)+6.49\right)}}$$ (2)

Lastly, growing degree days (GDD; unitless), a measure of overall temperature accumulation, were similarly calculated following equations and parameters from Cornell University’s Climate Smart Farming GDD calculator [40] (Eq. 3).

$$\mathrm{GDD}=\left\{\begin{array}{c} 0,\;\text{if}\;\frac{T{\left(t\right)}_{\max}\;+\;T{\left(t\right)}_{\min}}{2}\;<\;0\\ \frac{T{\left(t\right)}_{\max}\;+\;T{\left(t\right)}_{\min}}{2},\;\text{if}\;\frac{T{\left(t\right)}_{\max}\;+\;T{\left(t\right)}_{\min}}{2}\;>\;0 \end{array}\right.$$ (3)

### RSA phenotyping in 2D space

We designed thin growth pouches made with acrylic sheets and germination papers (Anchor Paper Co., St. Paul, Minnesota, USA) [41] such that root growth is visible and contrasts strongly with the germination paper in 2-dimensional space (2D) [13]. We then adapted this system to predict 3D root growth in forestry Deepots (Stuewe and Sons, Inc., Tangent, Oregon, USA).

A blue germination paper measuring 279.4 × 381 mm was enclosed by a single acrylic sheet (TAP Plastics, Seattle, WA, USA) measuring 279.4 × 381 × 3.175 mm and a black high-density polyethylene sheet (HDPE) measuring 279.4 × 381 mm, secured by binder clips to block root exposure to light (Fig. S1).

To set up, wetted germination papers were placed on the acrylic sheet, followed by positioning a plant on top. The roots were arranged to ensure adequate contact with the germination paper. Finally, the black HDPE sheet was fastened using 2 binder clips on top and 2 on the sides.

Germination papers were placed in a crate and stored on a greenhouse bench where they were misted every other day. The greenhouse temperature fluctuated between 19 and 21 °C and did not vary between runs. Changes in outdoor day length between runs were supplemented by automated greenhouse lamps and/or shade cloths to maintain a 14-h day length. When in the crates, germination papers were held at a 60° angle incident to the ground to promote root growth along the germination paper. This angle was maintained using a polyvinyl chloride frame for the germination papers to rest.

Each crate was filled with 8 l of one-eighth strength modified Hoagland’s #2 solution [42,43] (Table S2) and replaced every 7 d. The nutrient solution was buffered with 0.1 M KOH to pH 6.3-6.8, with care to avoid salt deposition. The bottom 64 mm of the germination papers were submerged in nutrient solution to ensure consistent distribution of water across the germination paper.

During the course of the January and February harvest dates, the leaves became infested with a species of leafhopper. While they did not cause fatal damage to the plants, growth may have been affected in some way and imaging was delayed substantially for these trials. Spinosad, neem oil, and manual removal were used to control the leafhopper population.

We imaged germination papers once every 7 d, starting with the loading date as T0. Images were taken on a Canon EOS Rebel SL3 (Canon USA, Inc, Melville, New York, USA). January time point 4 at 28 d was skipped due to risks of leafhopper spread.

Germination papers were imaged after removing the black HDPE sheet. Lighting was controlled using 2 studio lights placed perpendicular to the imaging stage such that the greatest contrast between the roots and blue germination paper was achieved (Fig. S2).

The images required semimanual processing with ImageJ (NIH, Bethesda, Maryland, USA; LOCI, University of Wisconsin, Madison, Wisconsin, USA) to separate background media from roots before features could be extracted [44]. Because the blue background in the germination papers is designed to strongly contrast with the root system, a simple series of contrast increases and YUV thresholds in ImageJ produced a segmented image. Further filtering of sections of background artifacts smaller than 2 pixels was done in RhizoVision, prior to feature extraction.

Following segmentation in ImageJ, the resulting images were analyzed in RhizoVision [1], an open-source software designed for various root image analyses. RhizoVision generates a suite of root traits (see Table; Seethepalli et al. [1,45]) depending on the architecture type being imaged. Median number of roots, total root length, network area, convex area, solidity, and average diameter were deemed key traits, as they were biologically relevant under the context of common crop ideotypes [46–48] and also had a strong loading with principal component 1 in a principal component analysis (Fig. S3). While these ideotypes are not defined for trees, their goal of resource use efficiency is widely applicable.

**Table.** Set of equations used to generate the parameters for CropRootBox.jl

Parameter name	Description	Unit	Equation
*l_b_*	Length of apical zone	cm	$l_{b}=\frac{\text{NumberRoo}t_{\min}}{\text{RootLengt}h_{\min}}\times \text{InverseRootOrder}$
*l_a_*	Length of basal zone	cm	$l_{a}=\frac{\text{RootLengt}h_{\max}-\text{RootLengt}h_{\text{median}}}{\left(\text{NumberRoo}t_{\max}\right)\left(\text{InverseRootOrder}\right)}$
*l_n_*	Length between lateral branches	cm	$l_{n}=\frac{\text{SurfaceAre}a_{\text{median}}}{100\left(\text{NumberRoo}t_{\text{median}}\right)}\times 5{\left(\text{RootOrder}\right)}^{2}$
*l_max_*	Maximal root length	cm	$l_{\max}=\frac{\text{Dept}h_{\max}}{12{\left(\text{RootOrder}\right)}^{3}}$
*r*	Initial elongation rate	cm d^−1^	$r=\frac{\left(\text{RootLengt}h_{\max}-\text{RootLengt}h_{\min}\right)}{t}\times \frac{1}{5\left(\text{RootOrder}\right)}$
*Δx*	Resolution along root axis	cm	∆*x_primary_* = 0.5 ∆*x_secondary_* = 0.1 ∆*x_tertiary_* = 0.1
*σ*	Standard deviation of random angular change	cm^−1^	*σ* = *StandardDeviation*(*AverageRootOrientation*)
*θ*	Insertion angle	°	*θ* = *AverageRootOrientation*
*N*	Number of trials (tropism strength)		*N* = *RootOrder*
*a_i_*	Initial radius	cm	$a_{i}=\frac{\text{Diamete}r_{\min}}{{\text{NumberRoot}}_{\min}}\times \frac{1}{{\left(\text{RootOrder}\right)}^{2}}$
*a_max_*	Maximum radius	cm	$a_{\max}=\frac{{\text{Diameter}}_{\max}}{5{\left(\text{RootOrder}\right)}^{2}}$
*a_r_*	Radius growth rate	mm h^−1^	$a_{r}=\frac{a_{\max}-a_{i}}{t}$

### Model development

This implementation of CropRootBox.jl [17] was adapted from CRootBox [16] and translated to Cropbox, a crop modeling framework written in Julia [17]. Each root segment is defined by a set of parameters supplied by the user and is visually represented in the 3D interface by a mesh in the GeometryBasics.jl [49] package. The final 3D rendering is done in the Makie.jl [50] package.

All model parameters were calculated using equations as combinations of RhizoVision traits [1] (Table). These equations were built initially from biological relevance between the traits and parameters and were tweaked as needed following visual comparison of the model outputs. See Table S1 for equation shorthand meanings.

The majority of these parameters were implemented as they are in each RootSegment, except for random angular change which was normalized to:

$$\sigma_{∆x}=\sqrt{∆x}\cdot \sigma$$ (4)

and controls random and directed changes in the root segment growth.

The initial direction of a lateral root is controlled by the insertion angle θ, drawn randomly from the user-defined mean and standard deviation, and a radial angle uniformly randomly chosen from 0° to 360°.

Each root segment also obeys real-world root growth characteristics such as plagiotropism, gravitropism, and exotropism rules.

Lastly, CropRootBox.jl, like CRootBox, is a stochastic model—each parameter is chosen from a truncated normal distribution and each simulation is therefore one of many possible combinations of a parameter set.

### Model evaluation in 3D space

CropRootBox.jl model outputs were evaluated using 5-fold cross validation steps from existing images (see Dynamic phenotypic analysis of N responses) as well as an additional sample set of *P. trichocarpa* cuttings planted in sand for root crown imaging, as performance evaluation for 3D structure simulation. Model outputs were also rerun through RhizoVision to compare feature extractions against the original features.

Additional cuttings were obtained from a mid-December harvest. These cuttings were allowed to grow roots ranging from 12 to 76 mm in length in a hydroponic solution before being transplanted into Deepots (Stuewe and Sons, Inc. D40H Heavyweight Deepot Cell) measuring 63.5 mm × 254 mm with a volume of 656 ml filled with silica sand. The cuttings were imaged prior to transplantation, similar to Fig. S2, to establish an initial time point for root growth. After transplantation, the Deepots were placed in a mist tent for 1 week to facilitate recovery before being moved to a greenhouse bench. Each plant received a weekly application of 50 ml of one-fourth strength Hoagland’s #2 solution.

Forty eight of 50 plants survived, half of which were harvested at 35 d, and the remaining half at 77 d, while ensuring an even distribution of initial root vigor. The sand was gently removed from the pot and roots, followed by a light mist to rinse off any remaining sand. The roots were then laid out uniformly on a black HDPE sheet, and any roots extending beyond a 10-cm radius from the stem were trimmed.

Similar to the germination paper material, the root crown material was affected by leafhoppers, and a similar treatment approach was implemented.

Root crowns were then hung from an imaging box derived from the RhizoVision Crown imaging box (Fig. S4) [45]. They were imaged at an angle such that the greatest amount of root area was visible from a head-on image. Images were then processed in ImageJ and features extracted from RhizoVision in a similar manner to the germination paper analysis.

### Statistical analysis

All statistical analyses were conducted in R version 4.1.2 using RStudio build 492.

We conducted a simple linear regression using the R base stats package [51] on measures of thermal time against T50, where the lowest mean squared error (MSE) and *P* value at 5% α determined which relationships were statistically significant.

One-way analyses of variance (ANOVAs) using the R base stats package were conducted on T50 and RSA data by trait [52]. These results, depending on the significance at 5% α were then compared pairwise by a post-hoc Tukey honestly significant difference test [52] at 5% α using the R base stats package. In this manner, we compared the slopes of simple linear regressions of each trait for each plant during the germination paper imaging series and the monthly interaction for each time point along the time series.

Model outputs’ features were subsampled using a 5-fold cross-validation approach [53]. Three folds were reserved for training and 2 folds for testing and every permutation of folds was tested. *t* Tests with a Bonferroni *P* value adjustment using the rstatix package [54] were performed to compare model versus observed values for each trait. Significant differences in each trait were deemed misclassifications within each fold. To further evaluate the results, features extracted from a mature RSA model output were similarly compared to features from crown root images using *t* tests with a Bonferroni *P* value adjustment [55]. We considered the percentage of correctly predicted traits the sensitivity rate, as calculated in a confusion matrix [56].

## Results

### Adaptation of germination paper system to vegetative cuttings

**Fig. 2.**
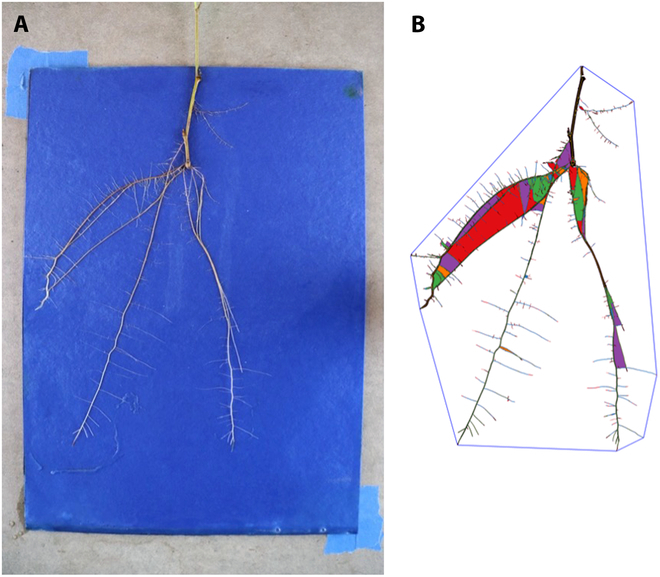
(A) Example adventitious root architecture 28 d after planting. (B) Image following feature extraction in RhizoVision. Filled colored areas represent regions of root overlap, or “holes”, and are a RhizoVision feature.

The seed-based germination paper system [13] is easily adaptable to a rooted vegetative cuttings and RhizoVision [1] features are able to be extracted through automatic batch processing (Fig. 2).

### Phenological effects on root system architecture

**Fig. 3.**
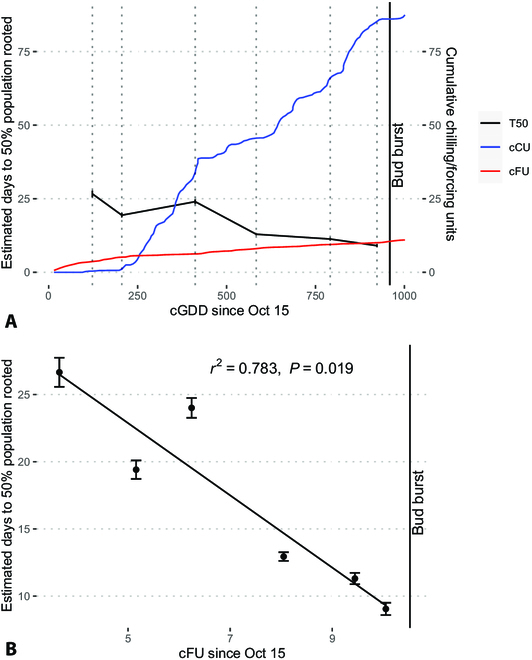
(A) Mean estimated time in days to 50% of a population of cuttings rooting in a hydroponic system (T50; black; primary y axis), plotted against cumulative growing degree days (cGDD) since October 15. Cumulative chilling units (cCU; blue; secondary y axis) and forcing units (cFU; red; secondary y axis) since October 15 are also plotted against T50. (B) Estimated time in days to 50% of a population of cuttings rooting in a hydroponic system (T50), plotted against cumulative forcing units (cFU). Each point is the estimated T50 and estimated standard error bars with a simple linear regression line.

The phenological effects on RSA vary depending on the type of quantification. For instance, the time in days for 50% of a population of cuttings to initiate roots (T50) slowly decreases over the course of the dormancy cycle, measured against the cumulative growing degree days (cGDD) from October 15 through March 15, from bud set to bud burst in the 2022 to 2023 dormancy cycle (Fig. 3A).

When considering the relationship between T50 before bud burst and cumulative forcing units (cFU), there is a strong observed linear relationship between the 2 parameters (Fig. 3B). The negative linear relationship is significant at 5% α (*P* value = 0.0061, *R*^2^ = 0.875, MSE = 5.42) and is the strongest correlation between T50 and a measure of thermal time.

**Fig. 4.**
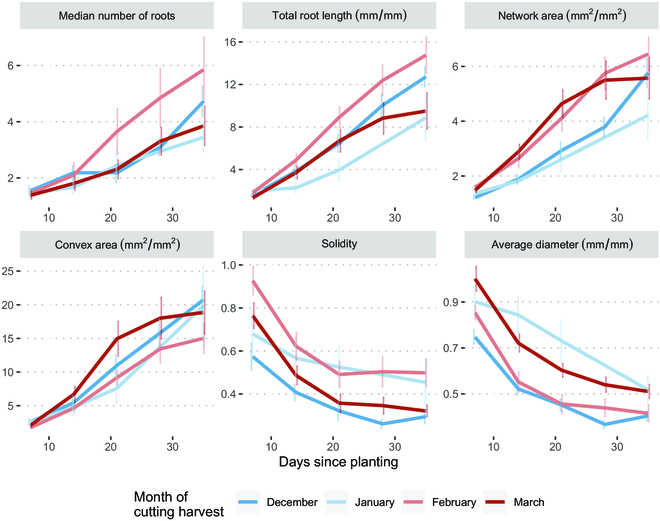
Time series of key RSA traits relativized to initial features (Tx/T0) extracted from RhizoVision by the time of cutting harvest. Each point represents the mean with standard error bars.

The RSA traits, when relativized by dividing each time point by its T0 value, exhibit no discernible differences in rooting across phenological stages (Fig. 4). Limited significant differences are observed between months at each time point, and no clear trends are evident. A 1-way ANOVA conducted on linear regressions for each plant’s time series for each key trait show no significant differences in slopes between months at 5% α (*P* value = 0.278) (Fig. S5).

**Fig. 5.**
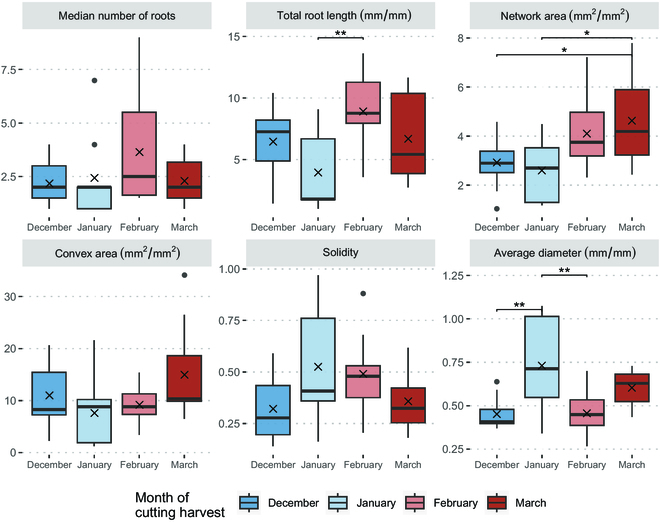
Boxplot of key RSA traits at 21 d since planting relativized to initial imaging (T3/T0). The “X” symbols indicate the mean of each dataset. Brackets with “*” indicate significant differences between groups. Nonsignificant differences are not displayed.

Looking specifically at 21 d since planting for each month, we observed few significant differences at 5% α following a 1-way ANOVA (*P* value = 0.0633). A post-hoc Tukey test reveals scattered differences between collection time points (Fig. 5). February RSA has more and longer roots, though this is not a trend supported by March RSA. There are indications of a trend in network area, with March having greater network area than both December and January, though this indication disappears by 35 d since planting, with no significance following a 1-way ANOVA (*P* value = 0.206) (Fig. S6).

### Root system model generation

**Fig. 6.**
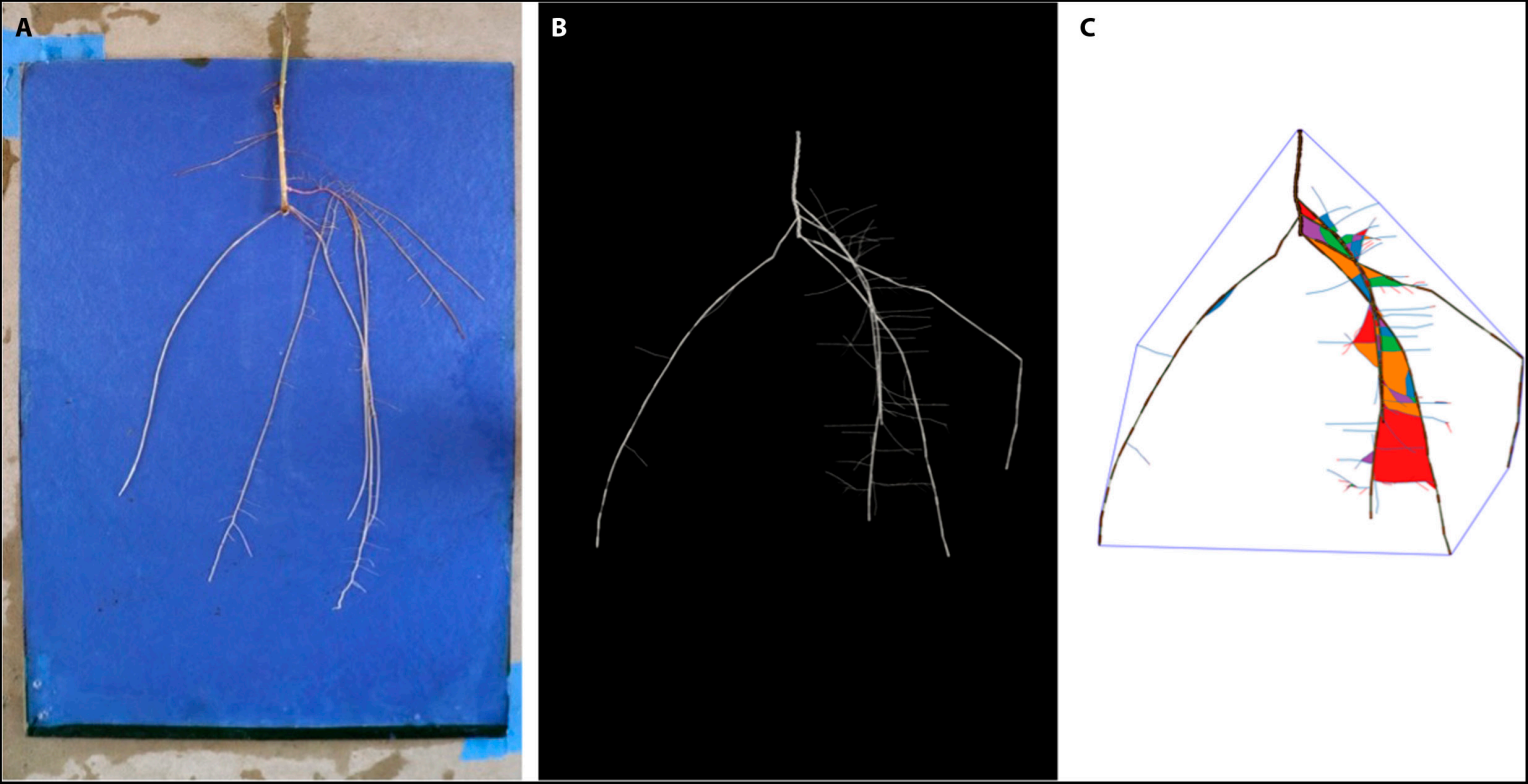
(A) Example adventitious root architecture used for parameterization. (B) Model simulation of RSA following parameterization from RSA images, not directly linked to (A). (C) Image following feature extraction of model output in RhizoVision.

Because there were no statistically significant trends found between phenology and mature RSA, we combined all the time-series images series into one full dataset and then subsampled from this to parameterize the model. Each model output was run through RhizoVision (Fig. 6).

**Fig. 7.**
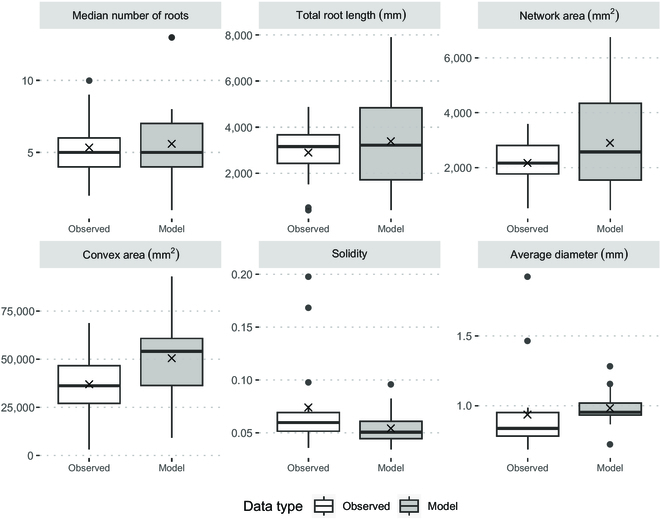
Comparison of 2D model output to observed 2D values of key RhizoVision traits in example fold. The “X” symbols indicate the mean of each dataset. None of the key traits had significant differences.

The features of the model outputs were then compared against the observed values in 2D in Fig. 7. From conducting a random sampling 5-fold cross-validation, with each significant difference in traits considered a misclassification, the average misclassification rate across 10 iterations was 6.4 of 39 measured traits, or a sensitivity rate of 83.5%.

**Fig. 8.**
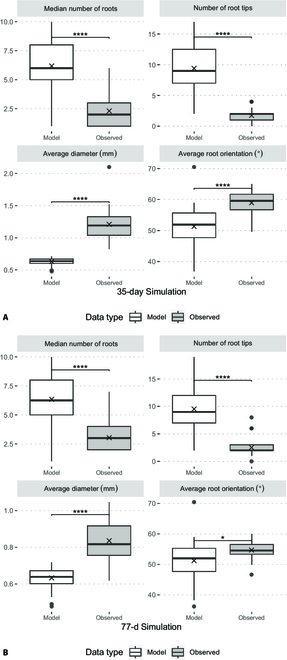
(A) Comparison of a 35-d 3D crown root simulation to true 3D crown root values of key RhizoVision traits. (B) Comparison of a 77-d 3D crown root simulation to true 3D crown root values of key RhizoVision traits. All the key traits had significant differences. The “X” symbols indicate the mean of each dataset. Brackets with “*” indicate significant differences between groups. Nonsignificant differences are not displayed.

Using this evaluated parameterization tool, we then compared model outputs of mature RSA simulated in 3D in a forestry Deepot against the true crown root features at a 35-d harvest and 77-d harvest. There were many reported significant differences among traits comparing the observed and model outputs. The 35-d simulation (Fig. S7A) had a misclassification rate of 20 of 39 traits, or a sensitivity rate of 48.7%, and the 77-d simulation (Fig. S7B) had a misclassification rate of 24 of 39 traits, or a sensitivity rate of 38.5%. The key traits for crown root analysis were all incorrectly predicted (Fig. 8).

## Discussion

We showed the viability of automatic parameter extraction and model evaluation for root FSPMs using time-series imaging and an application of RhizoVision. This overcomes the historical limitation of manual measurements and visual comparison in model parameterization. This system was also able to answer a meaningful question in the context of hardwood cultivation and management across the dormancy cycle, showing changes in the number of days until adventitious root initiation (T50), or the timing of the first root emergence, but not RSA, or the downstream development of the root system measured on germination papers.

### Germination paper system for vegetative cuttings

We were able to successfully adapt and simplify a germination paper system for use with vegetatively propagated cuttings. The material collection and maintenance methods were all conducive to usage with vegetative cuttings, though there were tradeoffs made when comparing to the original seed-based system.

For instance, the cuttings were harvested from trees impacted by regular waves of disease and pest damage and often harbored the same diseases and pests when brought into the greenhouse, which complicated the findings greatly. This problem is not present in seed germination [57,58] but can be avoided with thorough sterilization of both the cuttings and the germination papers. Additionally, because cuttings were transferred to germination papers following hydroponic root initiation, the imaged RSA may not accurately reflect the exact angles at which the roots were initiated. This problem is not present in seed-based germination paper RSA methods [13,57–59], where seeds are grown directly in the system and not initiated hydroponically. This may be circumventable in a future study by placing an unrooted cutting directly on the germination paper and would additionally allow for closer tracking of root initiation.

A benefit carried over from seed-based systems, regardless of transplanting, is ease of root sample collection [59], ease of assembly, and balance between proximity to natural conditions and resolution of data. The imaging and segmentation process was high-throughput, like in a seed-based germination paper system, with imaging and segmentation both taking roughly 1 min per germination paper. Because it is not time-intensive, we were able to regularly image and build time series. However, because this is a fully manual process, we would expect some decreases in efficiency the further we scale up this system. A system like GROWSCREEN-Rhizo [60], for example, is much more high-throughput because it is a fully automated process. However, it may be limited by lack of resources. In a field setting, a system such as minirhizotrons [61,62] would be necessary for the plants to grow in an outdoor environment though they lack more detailed information provided by a full RSA assay. Given all this, we determined that this phenotyping platform can be applied to most vegetatively propagated easy-to-root woody plants and meets our initial goal of being affordable, accessible, and high-throughput.

### The effects of dormancy phenology on adventitious RSA of vegetatively propagated *P. Trichocarpa*

Our new root phenotyping system was effective at tracking changes in adventitious RSA and root initiation over the course of the dormancy cycle. There is an observed decrease in the time it takes for roots to initiate in inverse correlation with forcing units. Previous literature has observed a parabolic relationship of *Populus* rooting ability with belowground GDD [34], which contrasts with our observed linear relationship with above ground forcing units. There are likely multiple potential mechanisms underlying the interactions between adventitious root initiation, cumulative forcing units, and the hormonal fluctuations present throughout the dormancy cycle. Many hardwoods, including *Populus* species, undergo a decrease in indole-acetic acid (IAA) and increase in abscisic acid (ABA) at bud set [63,64]. ABA has been shown to inhibit root growth while IAA stimulates it [25,65–67]. With bud set occurring at lower heat accumulation, this hormonal shift correlates with our observed decrease in T50 with fewer cumulative forcing units. The amount of nutrient stock available in the mother plant at the time of cutting may also have varied across the dormancy cycle, influencing the rate of root initiation in cuttings [25]. When studied further, this could potentially aid nurseries in their cutting collection timing and methodology in the context of a changing climate.

However, while there are strong indications of a relationship between root initiation and aboveground phenology (Fig. 2), there are few observed patterns with RSA. There are isolated differences in root traits among certain months, such as February having significantly greater number of roots (Fig. 3), but we cannot confidently attribute them solely to phenology when there were many external factors that could have influenced this. We speculate that any hormonal shifts present in the stem at the time of cutting would have balanced out later in the development cycle in the light- and temperature-controlled greenhouse. Because of this, we cannot assume that germination paper-grown RSA accurately reflects the RSA of outdoor nursery cuttings. Despite these limitations, studying RSA in response to cutting collection timing showed the strength of this germination paper system for time-series phenotyping.

### Automated feature extraction from root images to parameterize and evaluate a simple FSPM

We successfully developed a novel system of equations to translate RhizoVision features extracted from vegetatively propagated cuttings grown on germination paper to parameters needed to simulate root growth on a 2D plane in CropRootBox.jl. A high sensitivity rate of 83.5% is promising for future efforts with time-series RSA phenotyping and modeling. With most of this process being automated, extracting features and parameters from the time-series images was very high-throughput and offers future FSPMs a reliable source of parameterization. However, because the parameterization and the actual model simulations are in 2 different languages (i.e., R and Julia), there is room for improvements in efficiency through a more seamless integration. We additionally found that RhizoVision outputs are very sensitive to input settings and parameterization equations would need to be recalibrated for different RhizoVision settings.

Despite the accuracy in modeling roots on germination paper in 2D, there is a large dip in the accuracy when translating parameters from germination paper to 3D crown roots. This may be due to several functional limitations in the phenotyping process. The germination papers as a media have very low resistance to growth and high water holding capacity, whereas sand has a slightly higher resistance but low water holding capacity [68,69]. When translating these parameters to evaluate against sand-grown plants, there may be a large information loss as the roots are less restricted in their growth patterns in the germination paper [70]. The crown root imaging was also limited by a shorter growth time not allowing for full woody development of the roots. This led to the roots hanging incorrectly and not following the original structure.

Additionally, because the model was parameterized for vegetative cuttings, it may not accurately represent the RSA of other propagation methods. Studies in RSA of other hardwoods shows increased number of primary roots and lateral roots in vegetative cuttings compared to those of tissue culture and seed propagated plants [29,71]. Seed-grown plants especially have strong tendencies to develop a single taproot as opposed to multiple primary roots as in tissue culture and cutting propagation [29]. There are also strong differences in RSA among genotypes of herbaceous plants such as rice, reflecting their specific genotype by environment adaptations [72]. Model parameterization may need to shift to reflect this change in RSA across propagation methods and genotypes.

CropRootBox.jl, as a simplified version of CRootBox, demonstrates the ability of germination papers and RhizoVision in generating parameters for such models but does not represent the full strength of FSPMs. Missing features such as water movement and nutrient acquisition limit the functional aspects of the model, while the structural components are restricted by the implementation of parameters. Maximal root lengths, for example, cannot be surpassed by the model and lead to inaccuracy in simulations of greater length. Furthermore, a great strength of CRootBox not present in CropRootBox.jl is that it is coupled to aboveground processes in CPlantBox [73]. Water movement and maximal root lengths are coupled with aboveground processes, working beyond many of the limitations present in belowground-only FSPMs. However, combining automated parameterization with previous efforts in mature simulation [74] in CRootBox can easily supply parameters for more complicated models [19]. This tool, as an automated method for parameterization and model evaluation can help alleviate the difficulties of building root FSPMs and help work around the limitations of experimental RSA techniques.

### Conclusion

We successfully adapted a germination paper system to phenotype the RSA of adventitiously propagated *Populus trichocarpa* cuttings in a high-throughput manner. These time-series images allowed us to automate the parameterization process from raw images to model parameters, circumventing the error and lack of repeatability in manual parameterization methods. This process accurately predicted growth in germination papers but fell short when expanding from 2D to 3D model predictions. Despite the limitations in the model, the parameterization method is a strong first step in automatically generating parameters for other, more commonly used FSPMs.

## Data Availability

The model is housed in Github at github.com/uwkimlab/CropRootBox.jl_propagation.jl. All data and and R scripts are housed in Zenodo at https://doi.org/10.5281/zenodo.8083525.
