# Supplementary material for: Bridging Time-series Image Phenotyping and Functional–Structural Plant Modeling to Predict Adventitious Root System Architecture
Source: Plant Phenomics. 2023 Dec 21;5:0127. doi: 10.34133/plantphenomics.0127 (PMC10739341; doi:10.34133/plantphenomics.0127)
Supplement: Supplementary 1 — Figs. S1 to S7 Tables S1 to S2 [file plantphenomics.0127.f1.zip › Figure_S6.pdf]

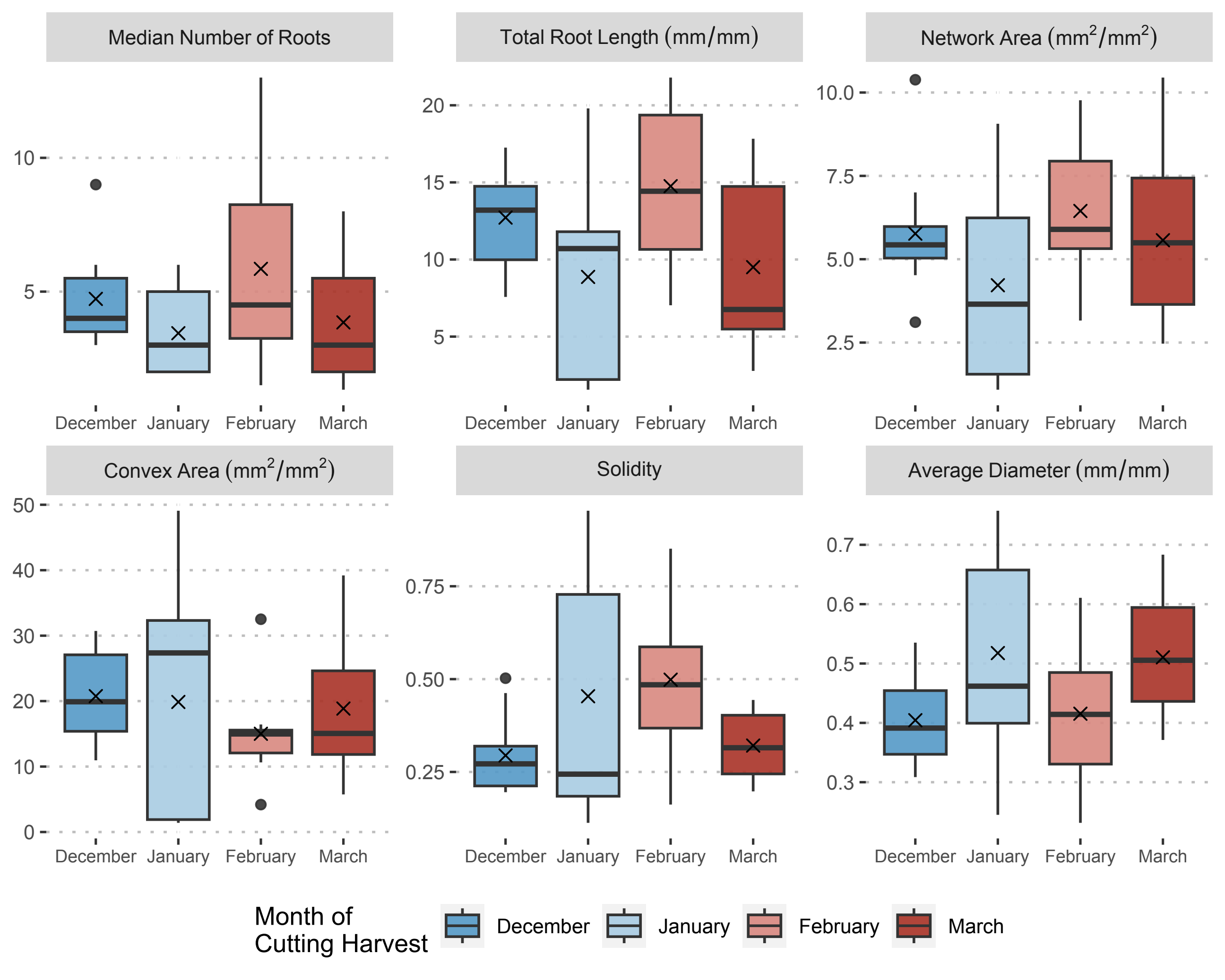

Median Number of Roots
Total Root Length (mm/mm)
Network Area (mm^2/mm^2)
Convex Area (mm^2/mm^2)
Solidity
Average Diameter (mm/mm)
December
January
February
March
Month of Cutting Harvest
