# Supplementary material for: Bridging Time-series Image Phenotyping and Functional–Structural Plant Modeling to Predict Adventitious Root System Architecture
Source: Plant Phenomics. 2023 Dec 21;5:0127. doi: 10.34133/plantphenomics.0127 (PMC10739341; doi:10.34133/plantphenomics.0127)
Supplement: Supplementary 1 — Figs. S1 to S7 Tables S1 to S2 [file plantphenomics.0127.f1.zip › Supplementary Materials.docx]

**Supplementary Materials**

Figures S1 to S7

Tables S1 to S2


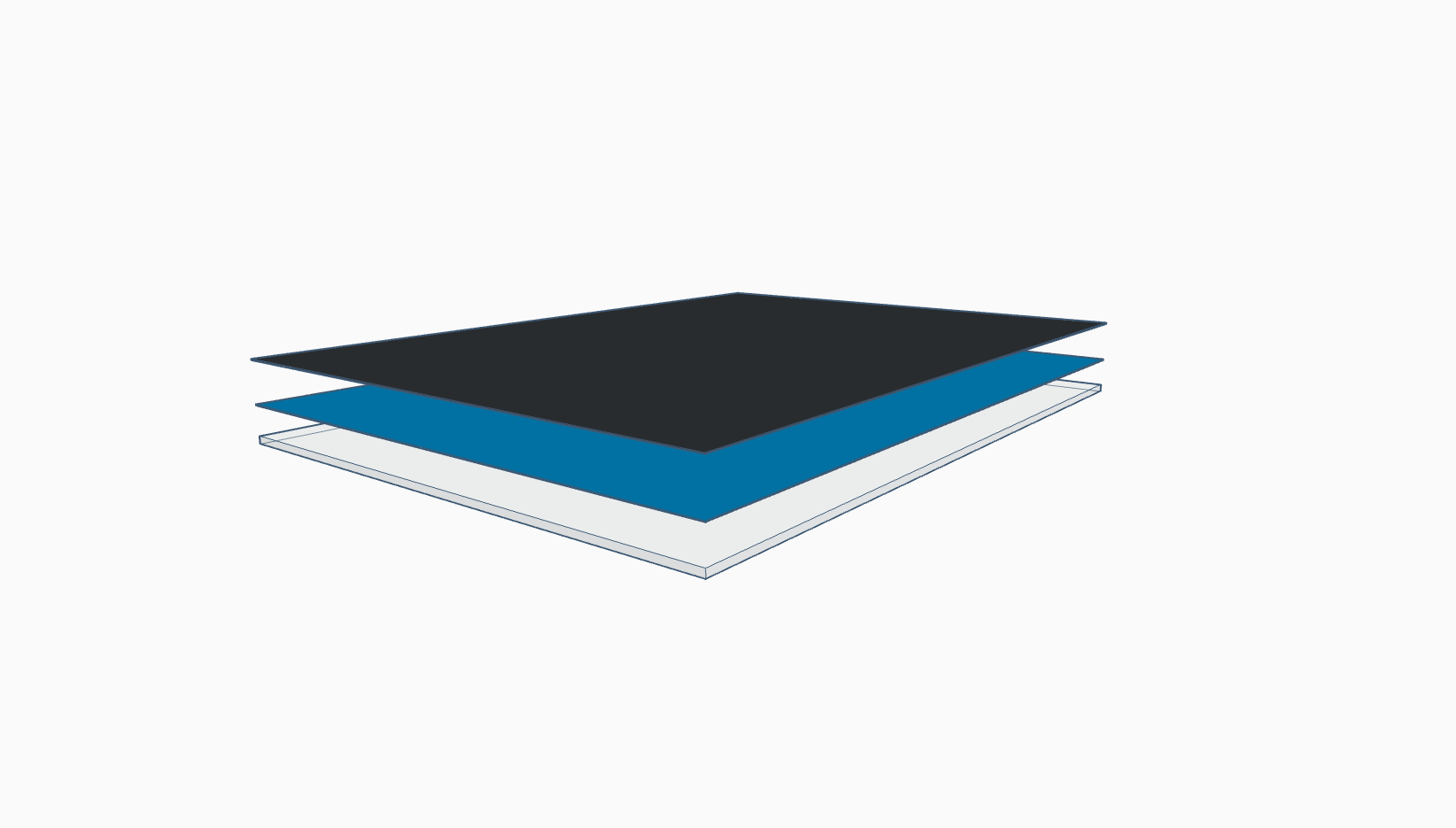


**(a)**


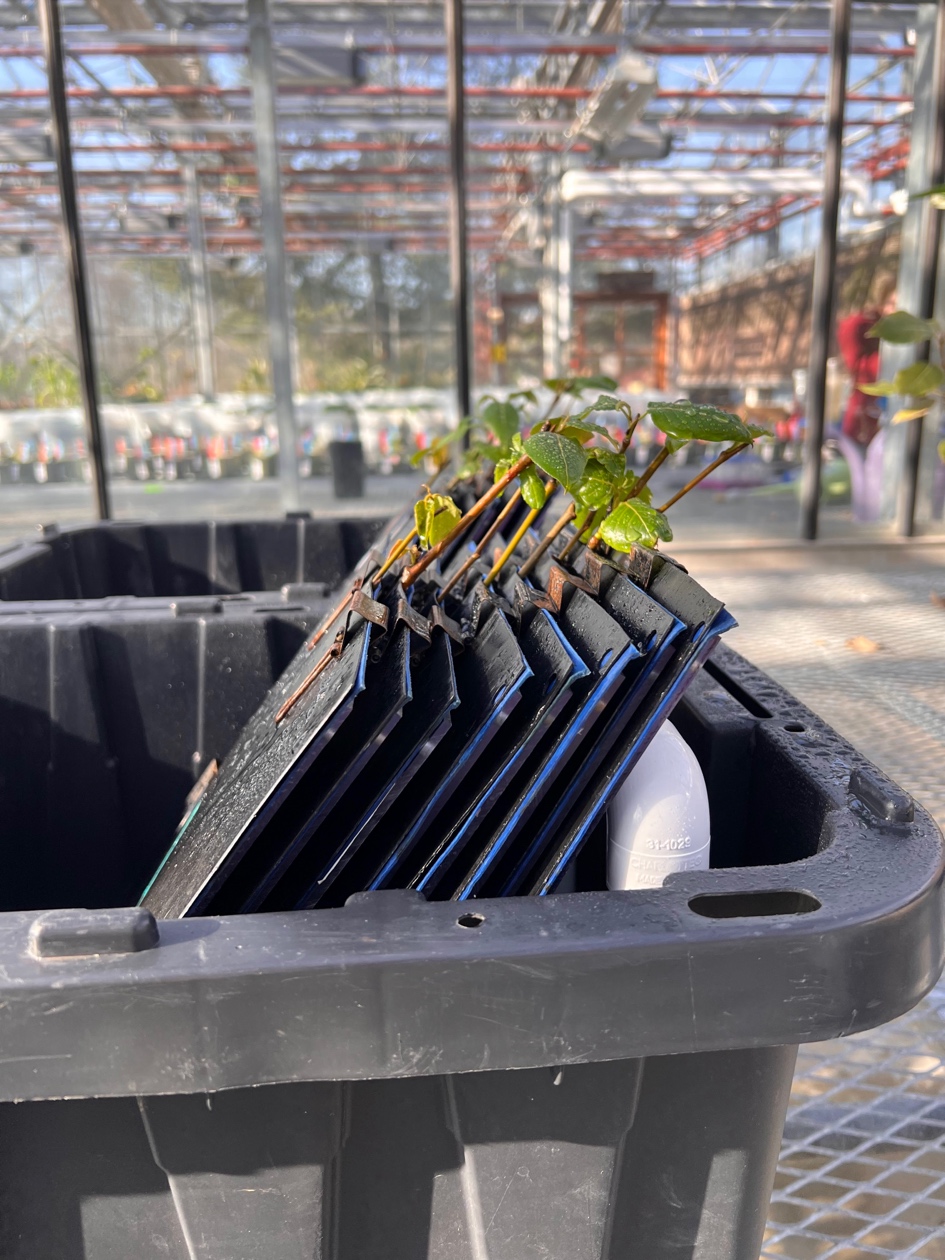


**(b)**

*Figure S1:* ***(a)*** *Schematic of germination paper assembly.* ***(b)*** *Image of multiple germination papers in a crate. Plants were sandwiched between the blue germination paper and black HDPE sheet.*

*
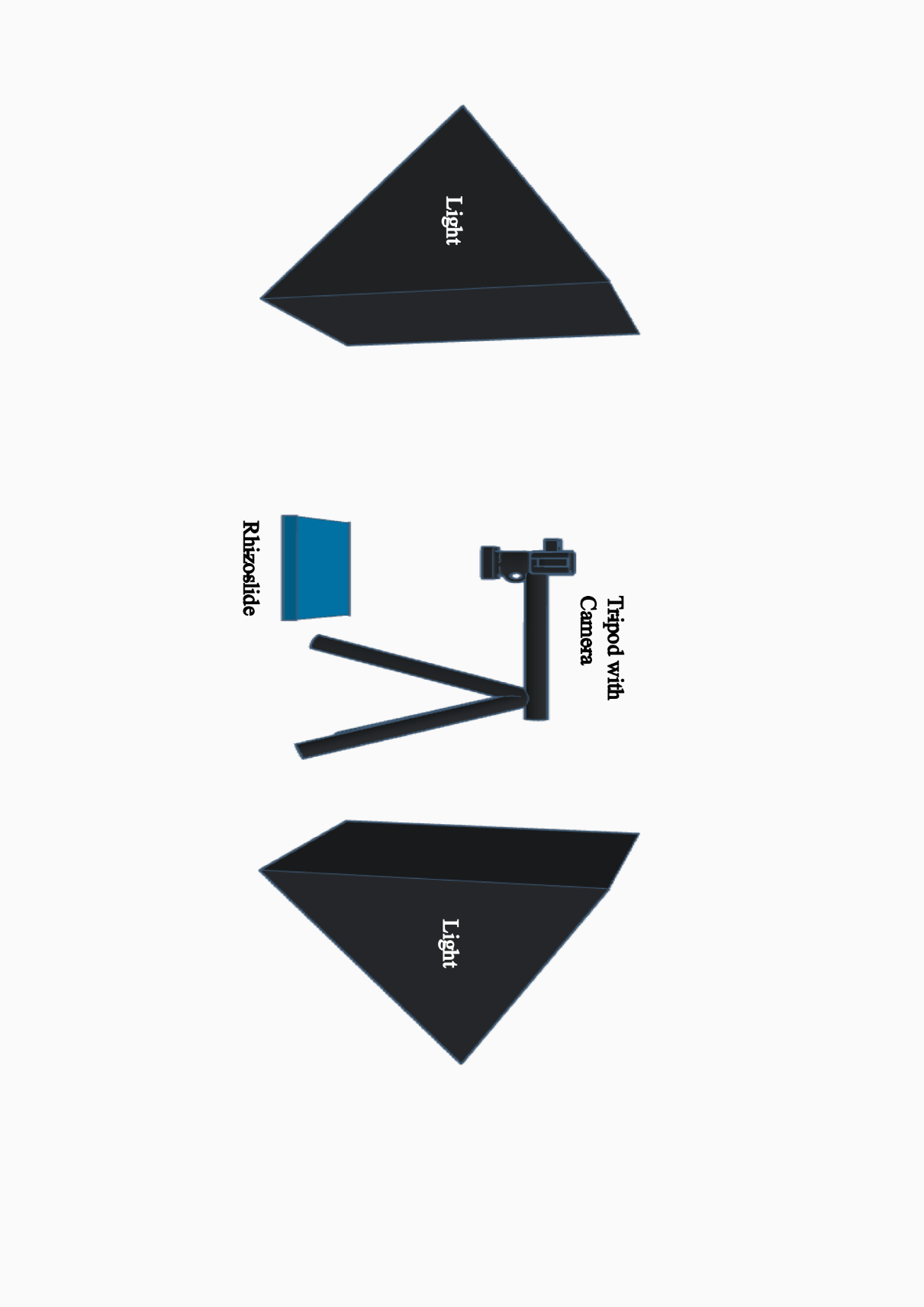
*

**(a)**


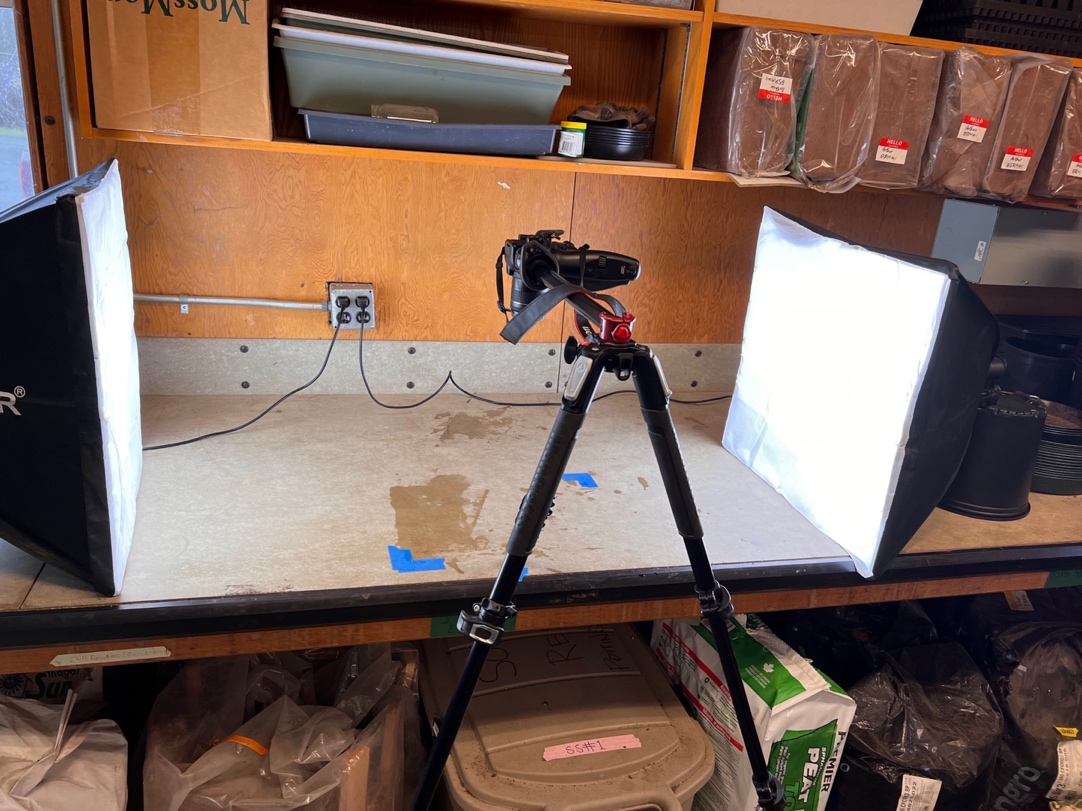


**(b)**

*Figure S2:* ***(a)*** *Schematic of the top-down lighting and imaging arrangement for germination paper.* ***(b)*** *Image of the lighting and imaging arrangement. The black HDPE sheet was carefully removed before imaging which allowed for head-on imaging. The camera was controlled remotely on a laptop using Canon’s EOS Utility 3.*

*
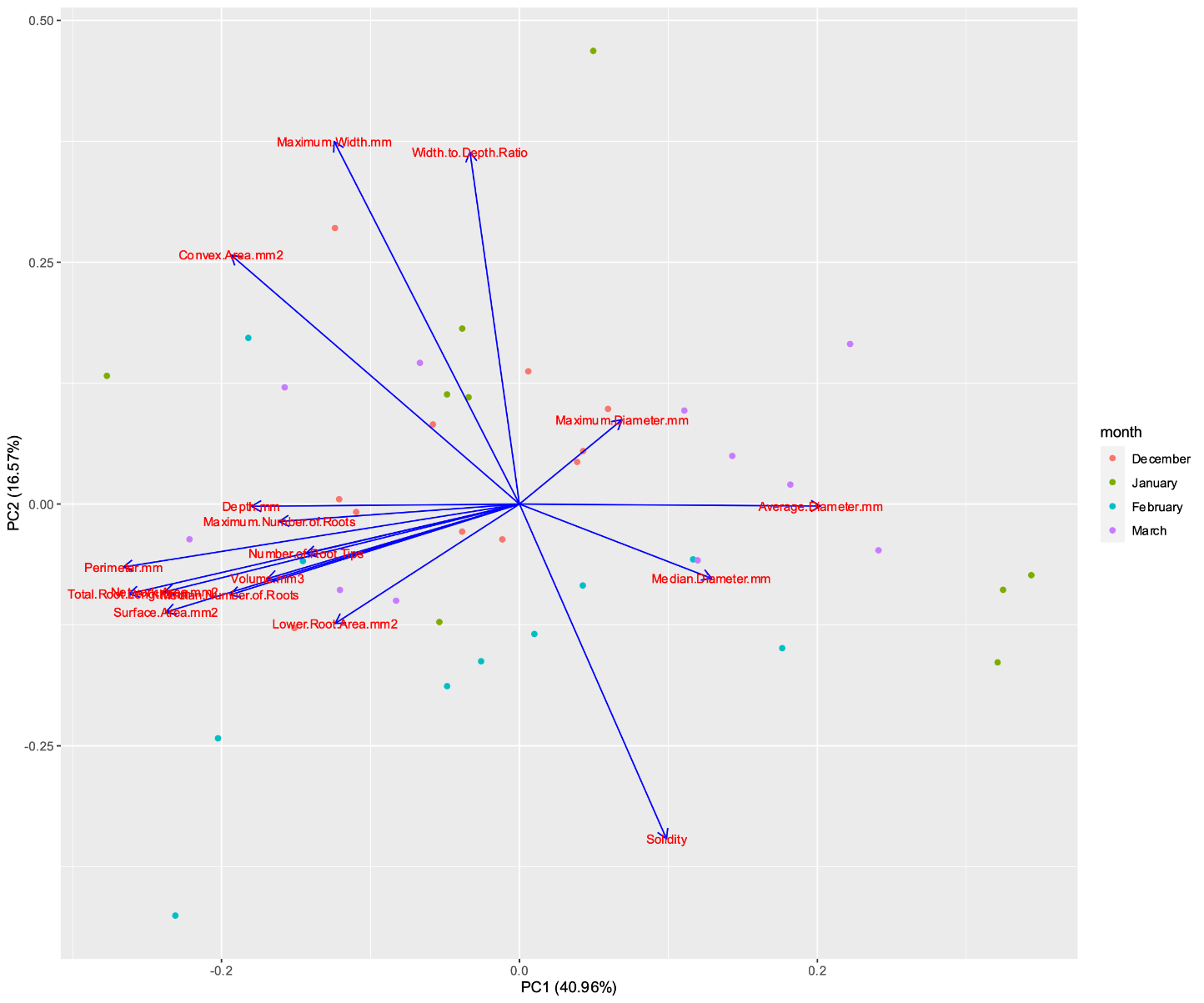
*

*Figure S3:* *Principal component analysis (PCA) of timepoint 5 at 35 days since planting, with trait loadings along PC1 and PC2. This PCA was used to inform which traits are key traits.*

*
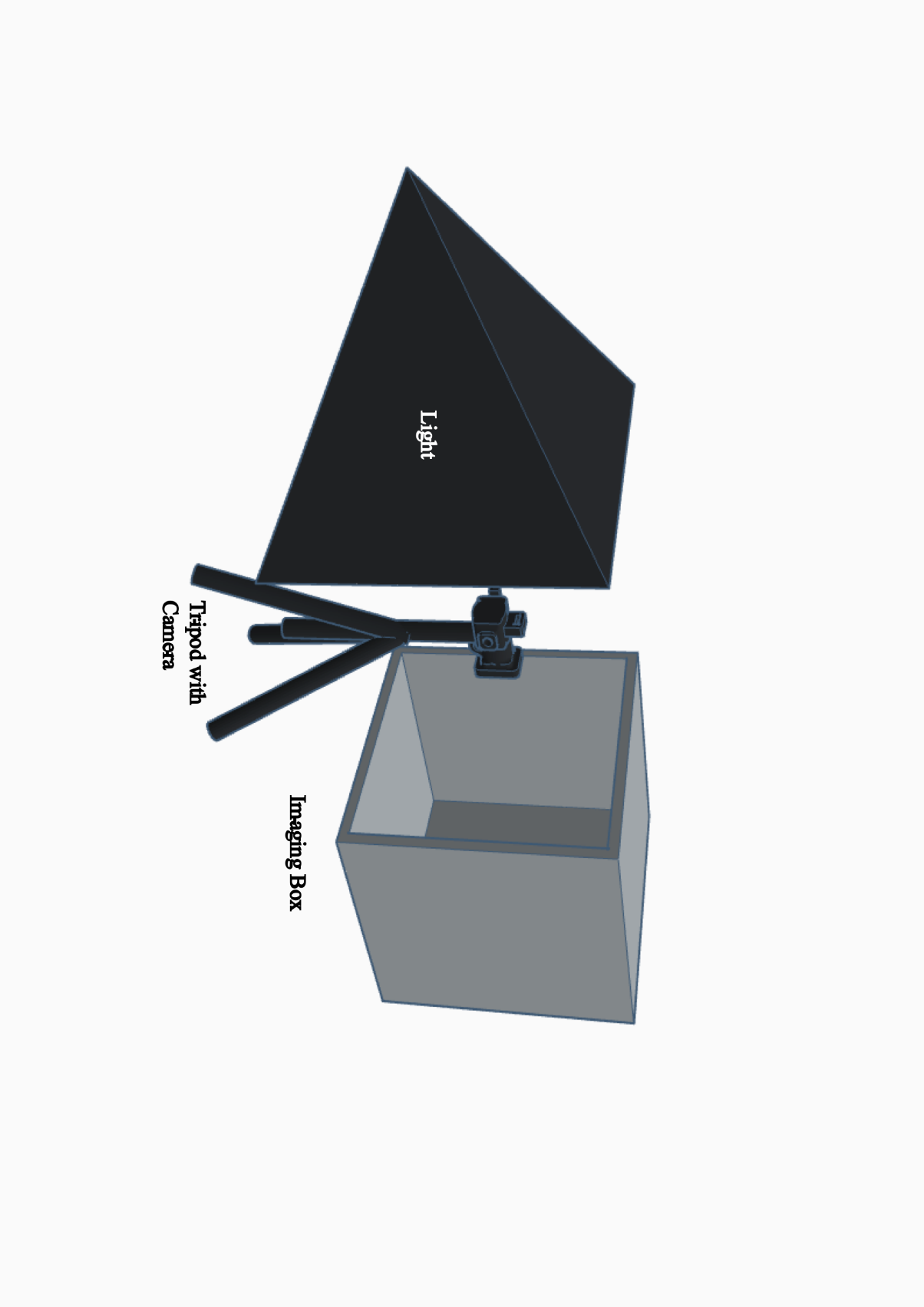
*

**(b)**

**(a)**


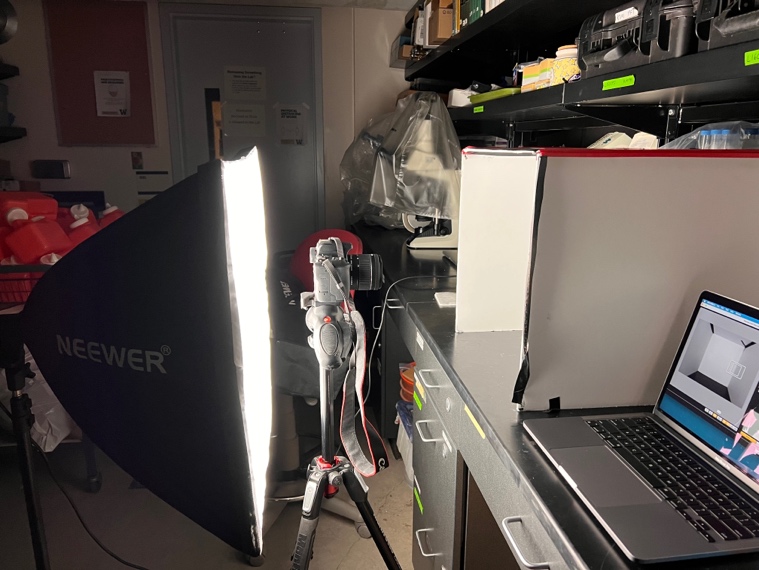


*Figure S4:* ***(a)*** *Schematic of the lighting and imaging arrangement for crown root material.* ***(b)*** *Image of the lighting and imaging arrangement. The plants were suspended from the hole in the foam imaging box. A studio light placed directly behind the camera in addition to a fully dark room created a strong contrast for the resulting crown root images. The camera was remotely controlled from a laptop using Canon’s EOS Utility 3.*

*
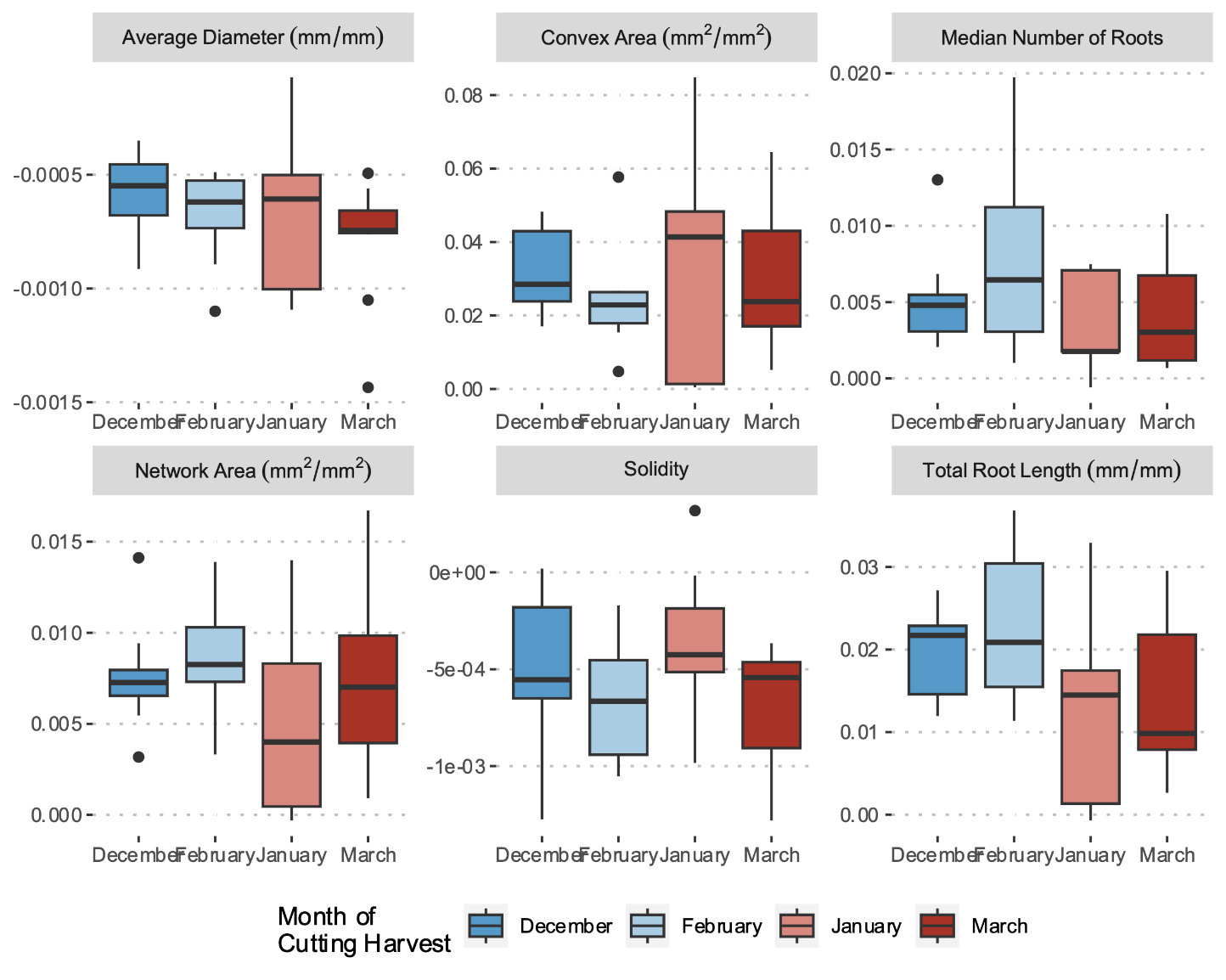
*

*Figure S5: Faceted boxplots of slope of linear regressions of individual plants’ time courses. No significant differences between slopes were found.*

*
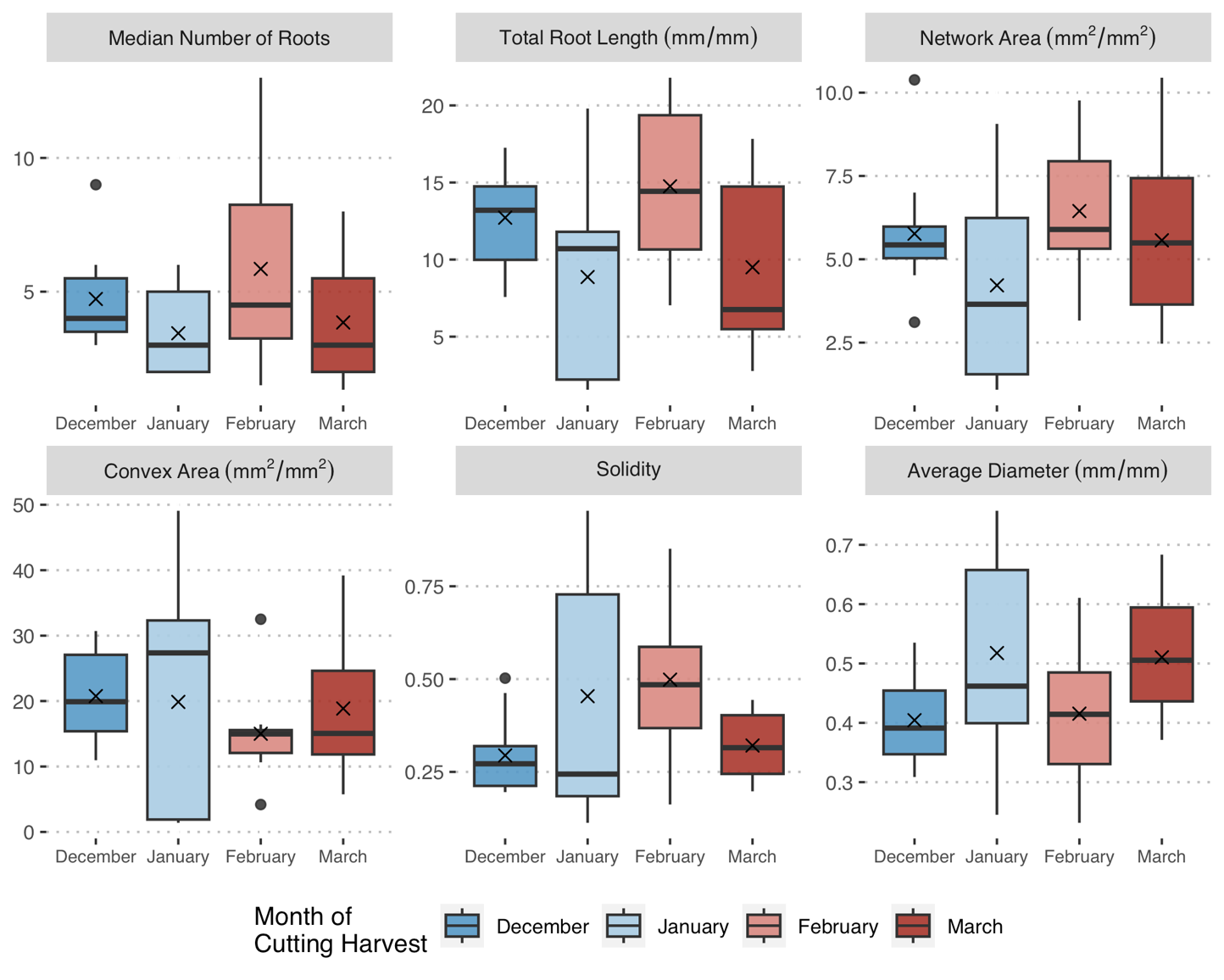
*

*Figure S6: Boxplot of key RSA traits at 35 days since planting relativized to initial imaging (T5/T0). The X symbols indicate the mean of each dataset.*

*
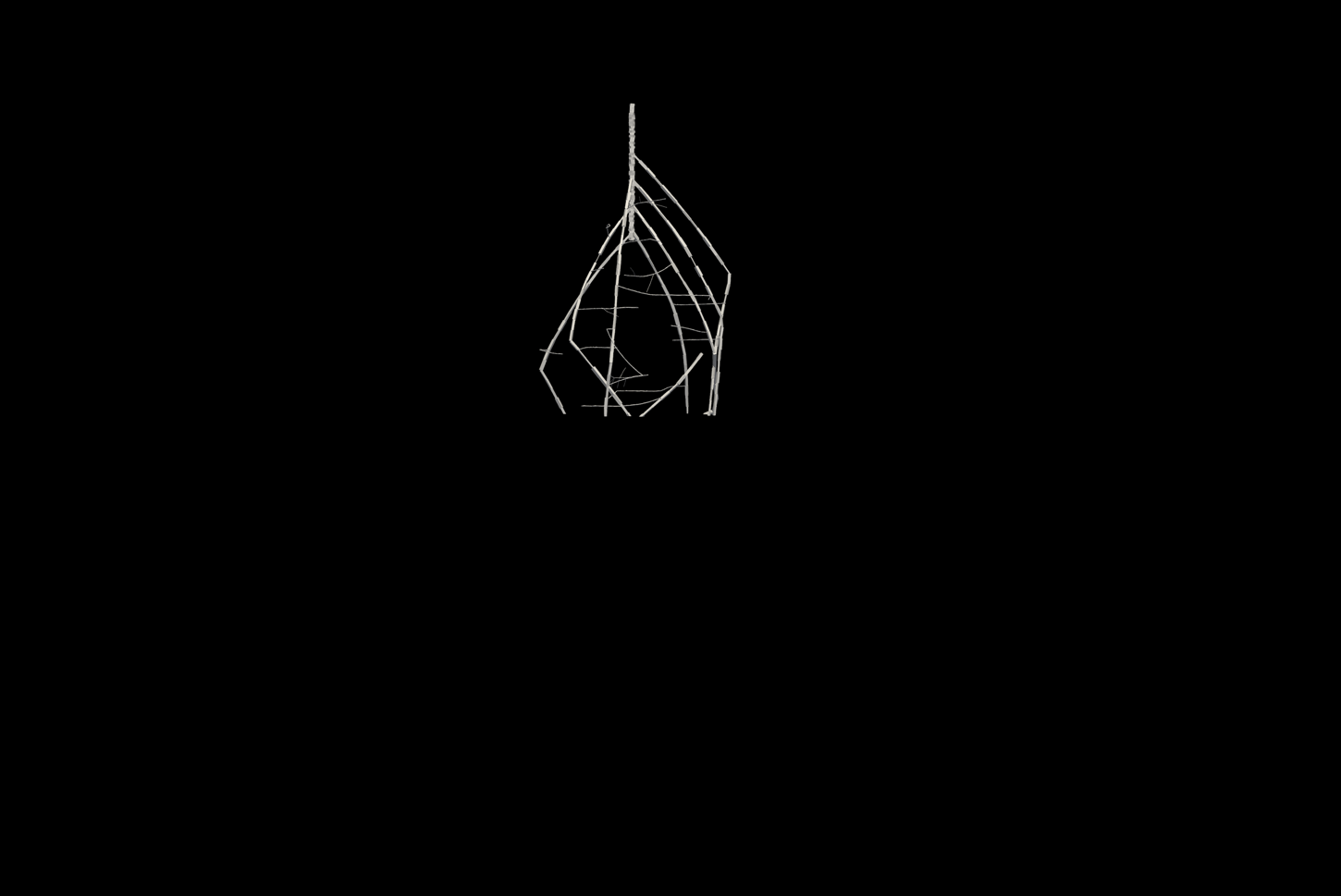

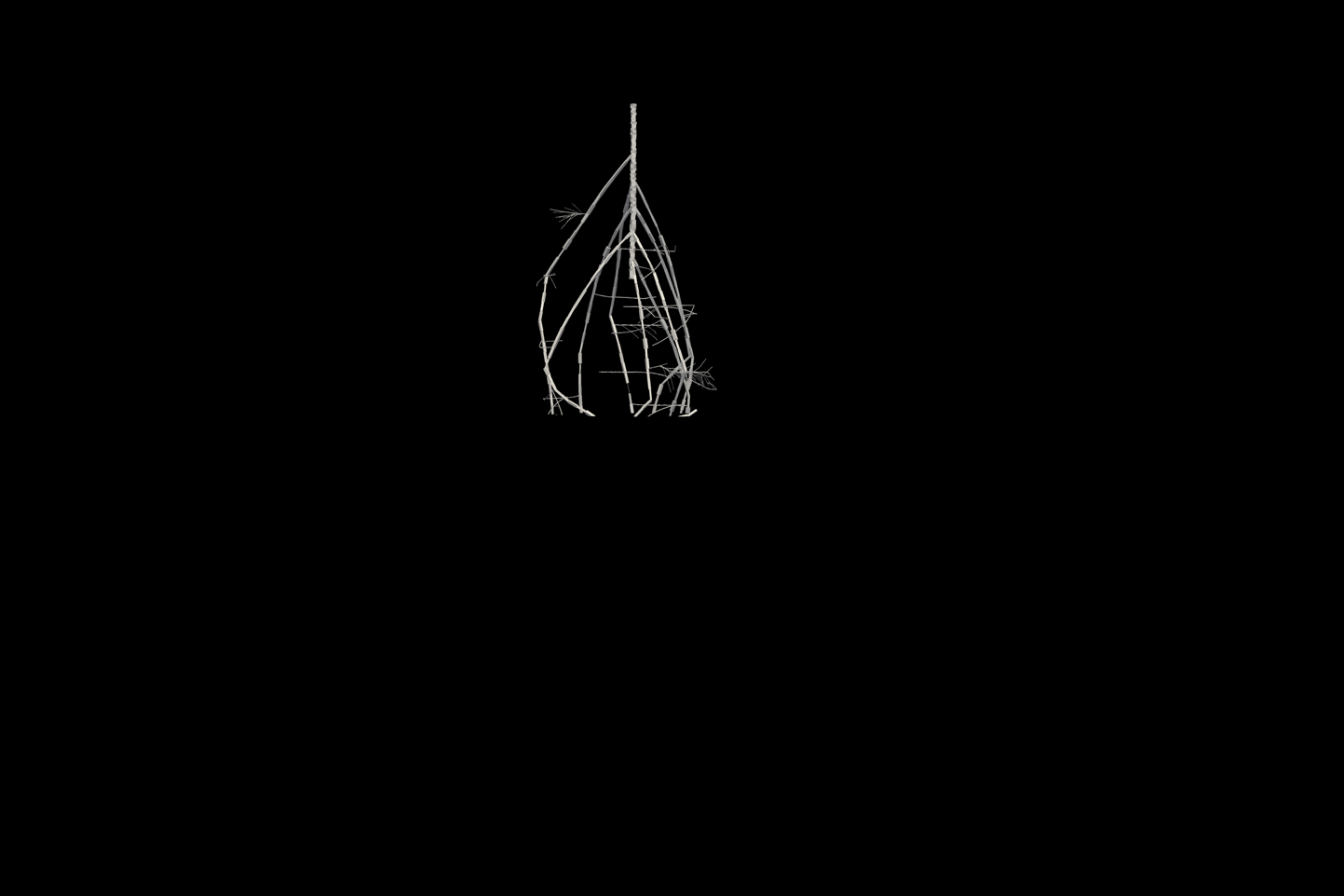
*

**(a)**

**(b)**

*Figure S7:* ***(a)*** *A 35 day model simulation of the crown root architecture used in model evaluation.* ***(b)*** *A 77 day model simulation of the crown root architecture used in model evaluation.*

*Table S1: Table 1 equation shorthands and their meanings.*

| **Equation Shorthand** | **Meaning** |
| --- | --- |
| (Inverse)RootOrder | Primary = 1, Secondary = 2, Tertiary = 3.  If inverse, Primary = 3, Secondary = 2, Tertiary = 1 |
| NumberRoot | RhizoVision Number of Roots |
| RootLength | RhizoVision Root Length by Diameter Range (mm) |
| SurfaceArea | RhizoVision SurfaceArea by Diameter Range (mm^2^) |
| Depth | RhizoVision Depth (mm) |
| AverageRootOrientation | RhizoVision Average Root Orientation (deg) |
| Diameter | RhizoVision Diameter (mm) |

*Table S2: Modified Hoagland’s solution recipe.*

| **Hoagland solution #2** (Epstein and Bloom, 2005) | |  | Strength: | **Full** | **1/2** | **1/4** | **1/8** | **1/16** | Stoc conc. |
| --- | --- | --- | --- | --- | --- | --- | --- | --- | --- |
| **Macronutrients** |  | MW | g/l (stock) | ml/L | ml/L | ml/L | ml/L | ml/L | mM |
| Potassium Nitrate | KNO3 | 101.1 | 101.1 | 6 | 3 | 1.5 | 0.75 | 0.375 | 1000 |
| Calcium Nitrate Tetrahydrate | Ca(NO3)2* 4H2O | 236.16 | 236.16 | 4 | 2 | 1 | 0.5 | 0.25 | 1000 |
| Ammonium phosphate monobasic | NH4H2PO4 | 115.08 | 115.08 | 2 | 1 | 0.5 | 0.25 | 0.125 | 1000 |
| Magnesium Sulfate Heptahydrate | MgSO4 * 7H2O | 246.49 | 123.245 | 2 | 1 | 0.5 | 0.25 | 0.125 | 500 |
|  |  |  |  |  |  |  |  |  |  |
| **Micronutrients** |  |  | total: | 2 | 1 | 0.5 | 0.25 | 0.125 |  |
| Potassium Chloride | KCl | 74.55 | 1.86375 |  |  |  |  |  | 25 |
| Boric acid | H3BO3 | 61.83 | 0.772875 |  |  |  |  |  | 12.5 |
| Manganese Sulfate (Mono)Hydrate | MnSO4 * H2O | 169.01 | 0.16901 |  |  |  |  |  | 1 |
| Zinc Sulfate | ZnSO4 * 7H2O | 287.54 | 0.28754 |  |  |  |  |  | 1 |
| Cupric sulfate | CuSO4 * 5H2O | 249.68 | 0.06242 |  |  |  |  |  | 0.25 |
| Molybdic acid | H2MoO4 (85% MoO3) | 161.97 | 0.040493 |  |  |  |  |  | 0.25 |
|  |  |  |  |  |  |  |  |  |  |
| Iron (Seq Fe 330) | NaFeDTPA (10% Fe) | 468.2 | 30 | 0.5 | 0.25 | 0.125 | 0.0625 | 0.03125 | 53.7 |
