# Supplementary figures and images for: Bridging Time-series Image Phenotyping and Functional–Structural Plant Modeling to Predict Adventitious Root System Architecture
Source: Plant Phenomics. 2023 Dec 21;5:0127. doi: 10.34133/plantphenomics.0127 (PMC10739341; doi:10.34133/plantphenomics.0127)

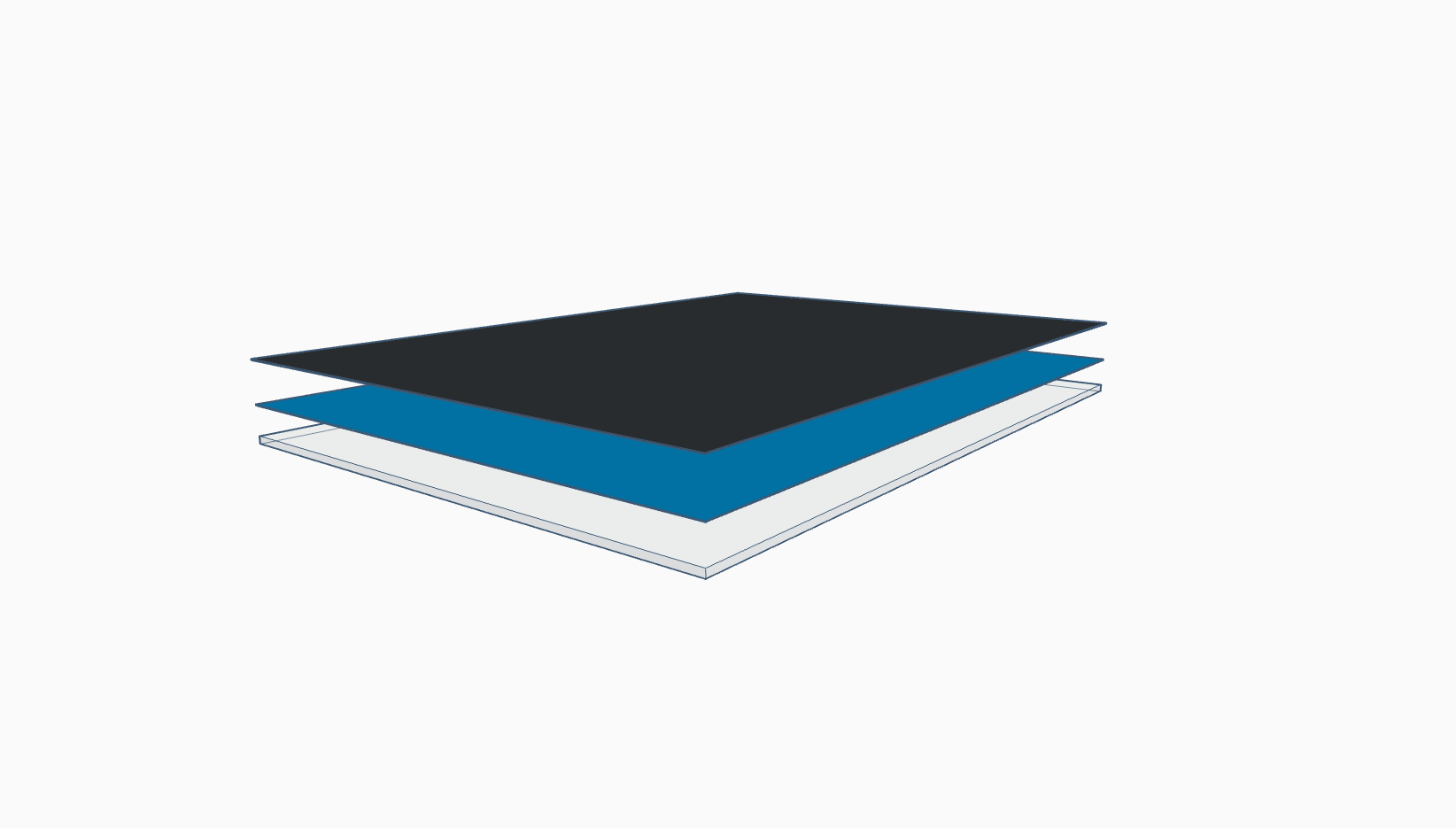

Supplement: Supplementary 1 — Figs. S1 to S7 Tables S1 to S2 [file plantphenomics.0127.f1.zip › Figure_S1a.png]

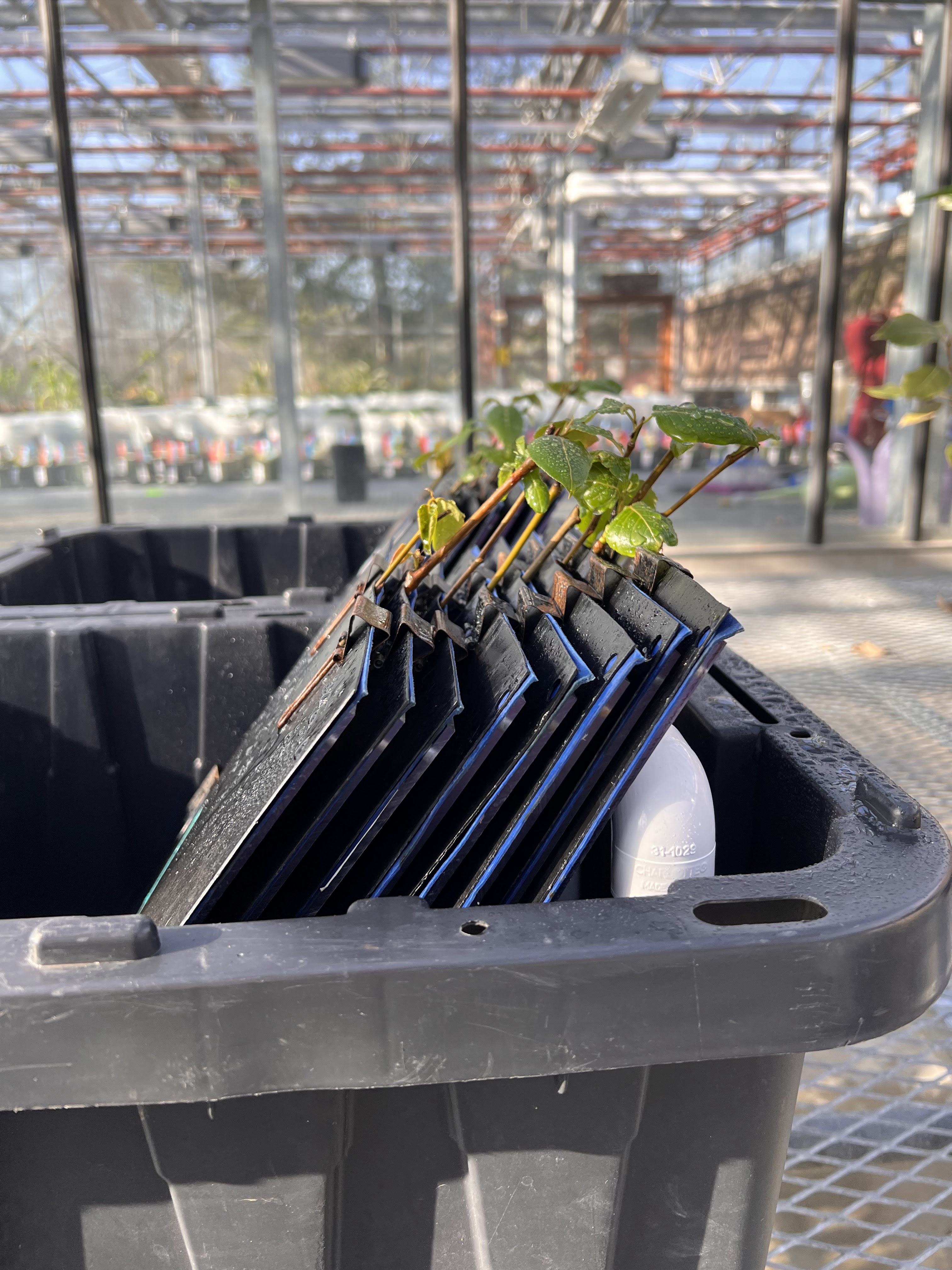

Supplement: Supplementary 1 — Figs. S1 to S7 Tables S1 to S2 [file plantphenomics.0127.f1.zip › Figure_S1b.jpg]

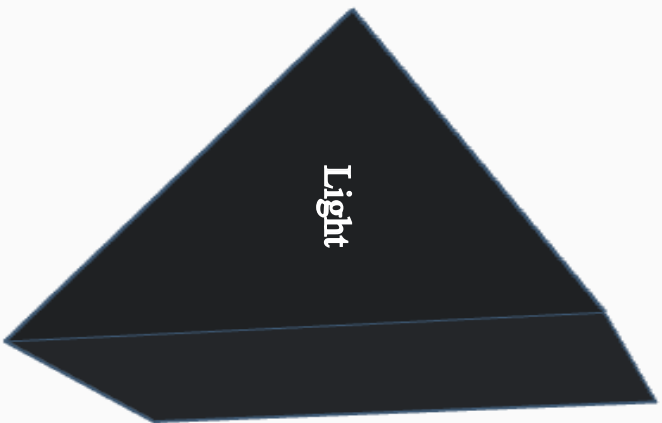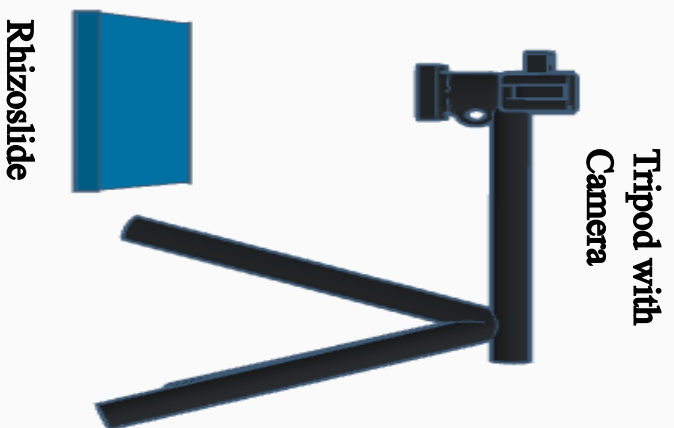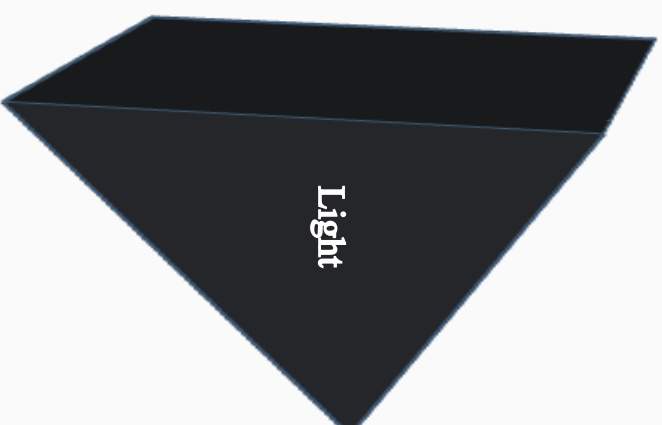

Supplement: Supplementary 1 — Figs. S1 to S7 Tables S1 to S2 [file plantphenomics.0127.f1.zip › Figure_S2a.pdf]

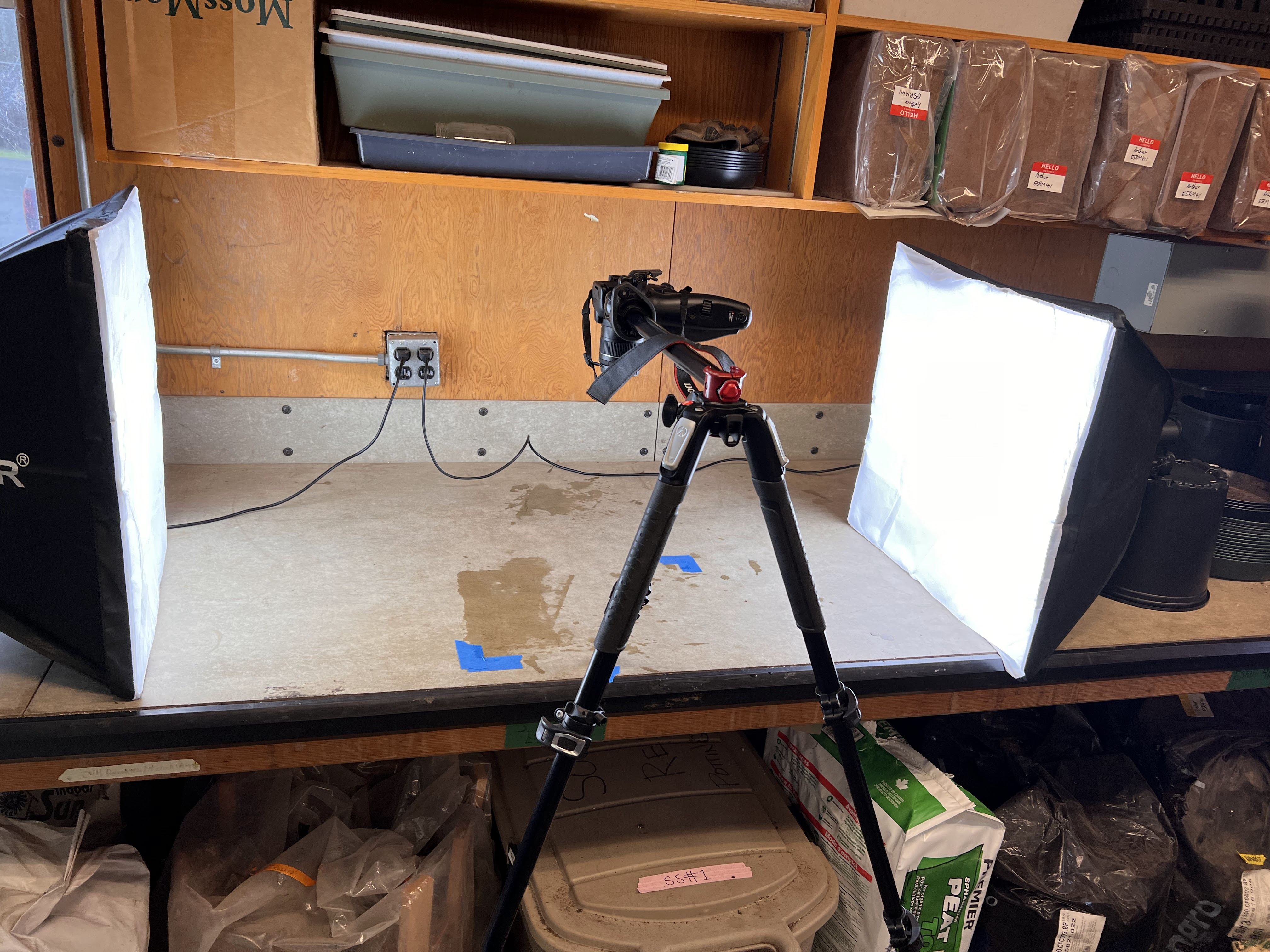

Supplement: Supplementary 1 — Figs. S1 to S7 Tables S1 to S2 [file plantphenomics.0127.f1.zip › Figure_S2b.jpg]

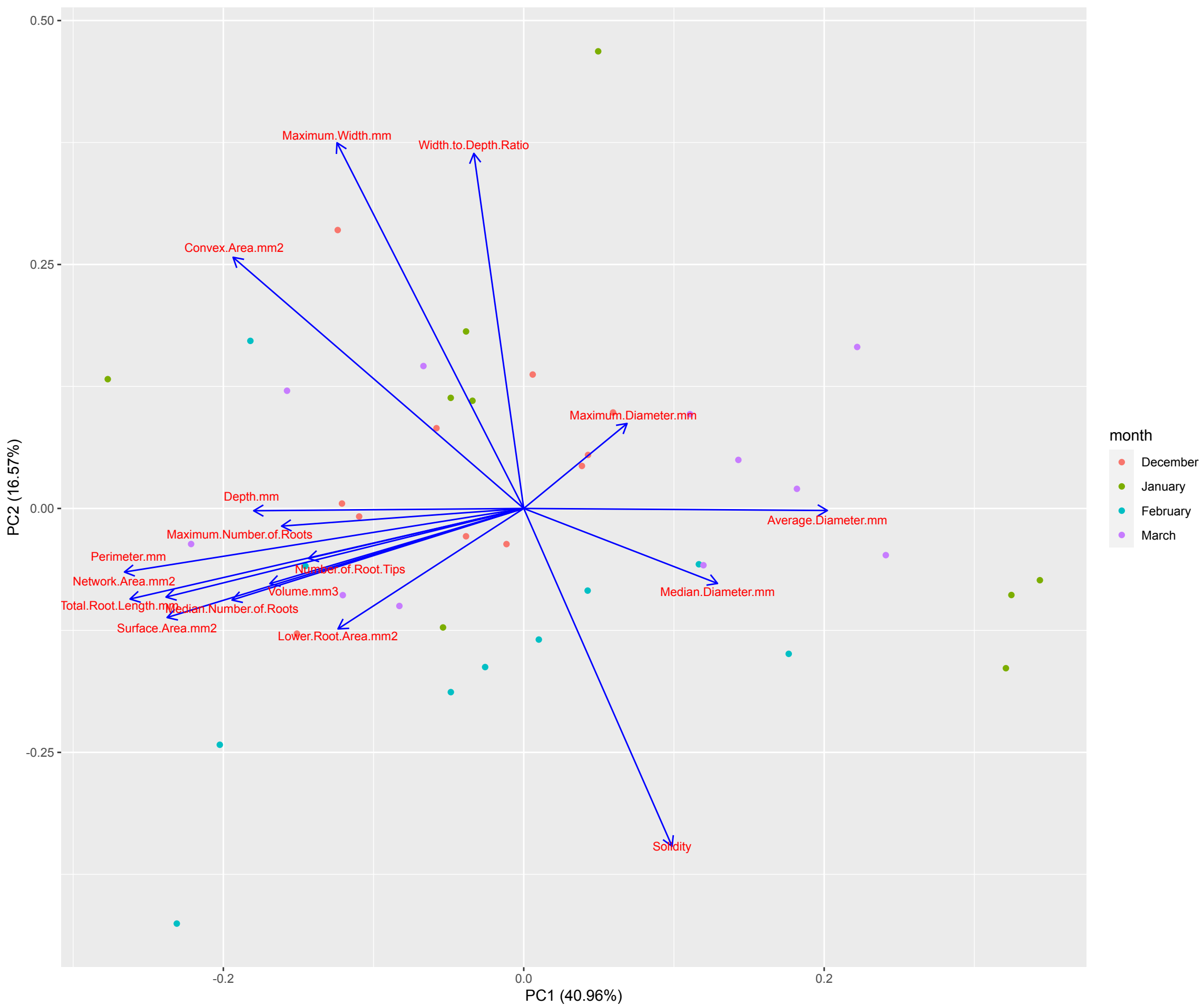

Supplement: Supplementary 1 — Figs. S1 to S7 Tables S1 to S2 [file plantphenomics.0127.f1.zip › Figure_S3.pdf]

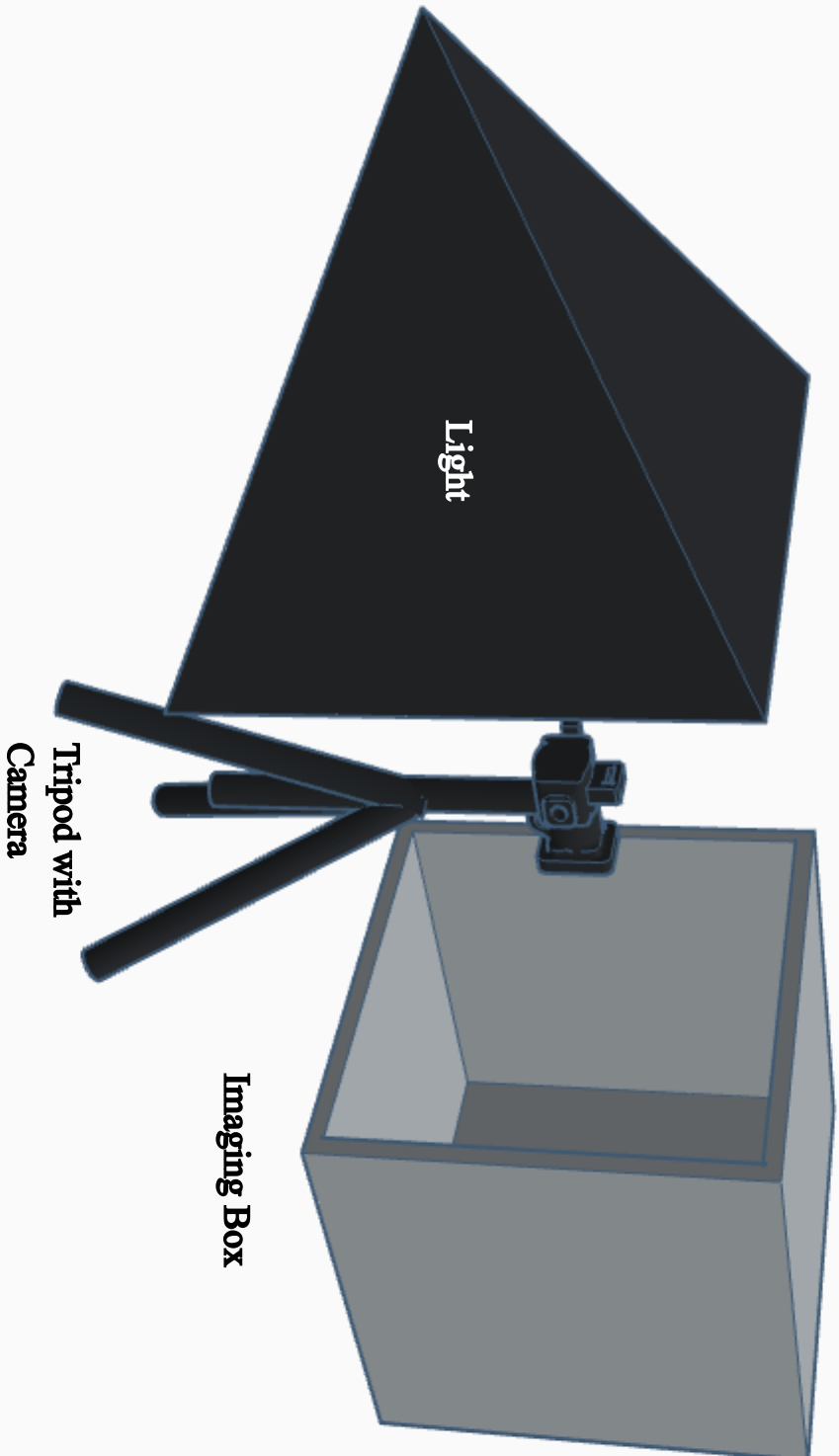

Supplement: Supplementary 1 — Figs. S1 to S7 Tables S1 to S2 [file plantphenomics.0127.f1.zip › Figure_S4a.pdf]

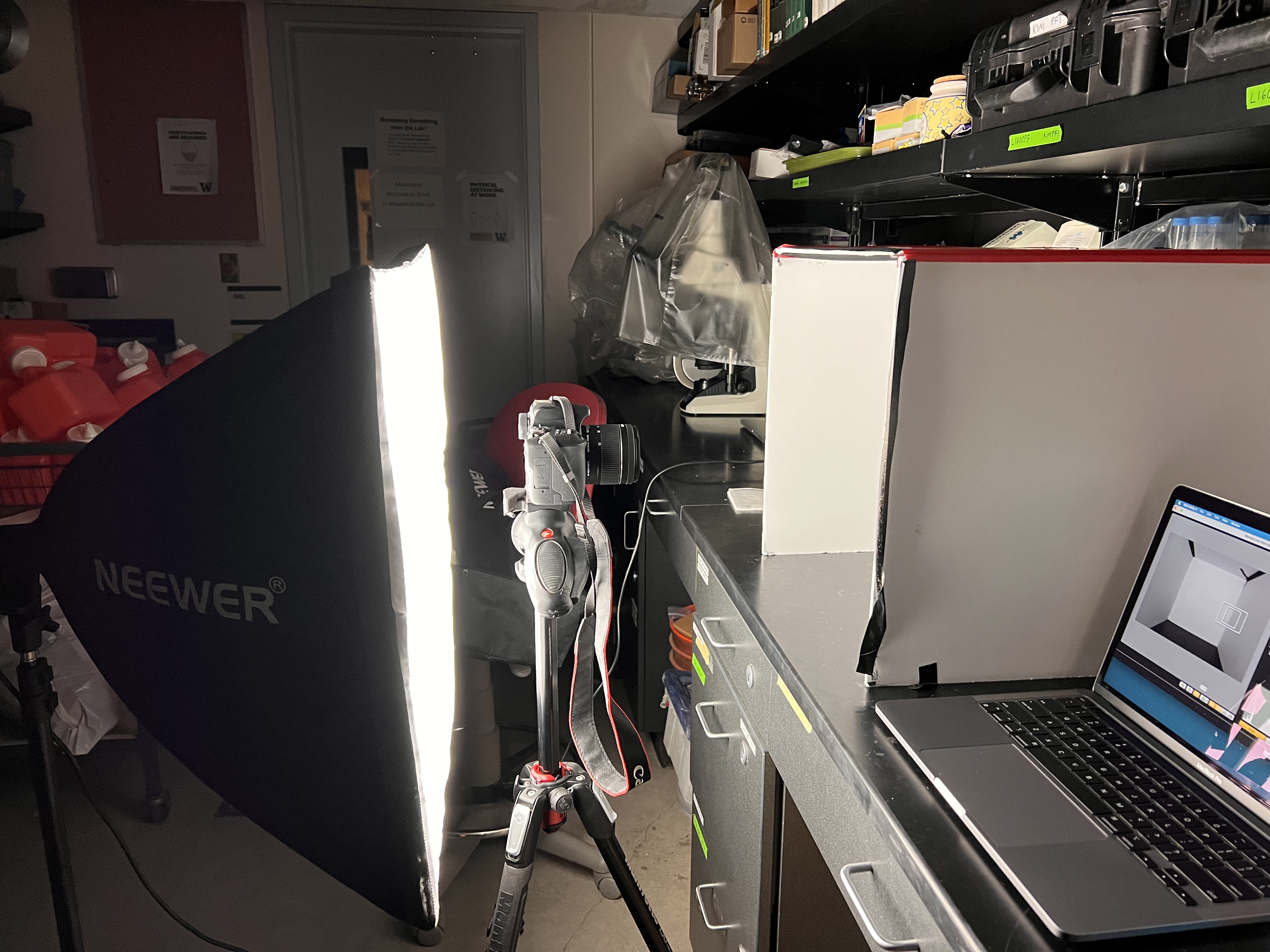

Supplement: Supplementary 1 — Figs. S1 to S7 Tables S1 to S2 [file plantphenomics.0127.f1.zip › Figure_S4b.JPG]

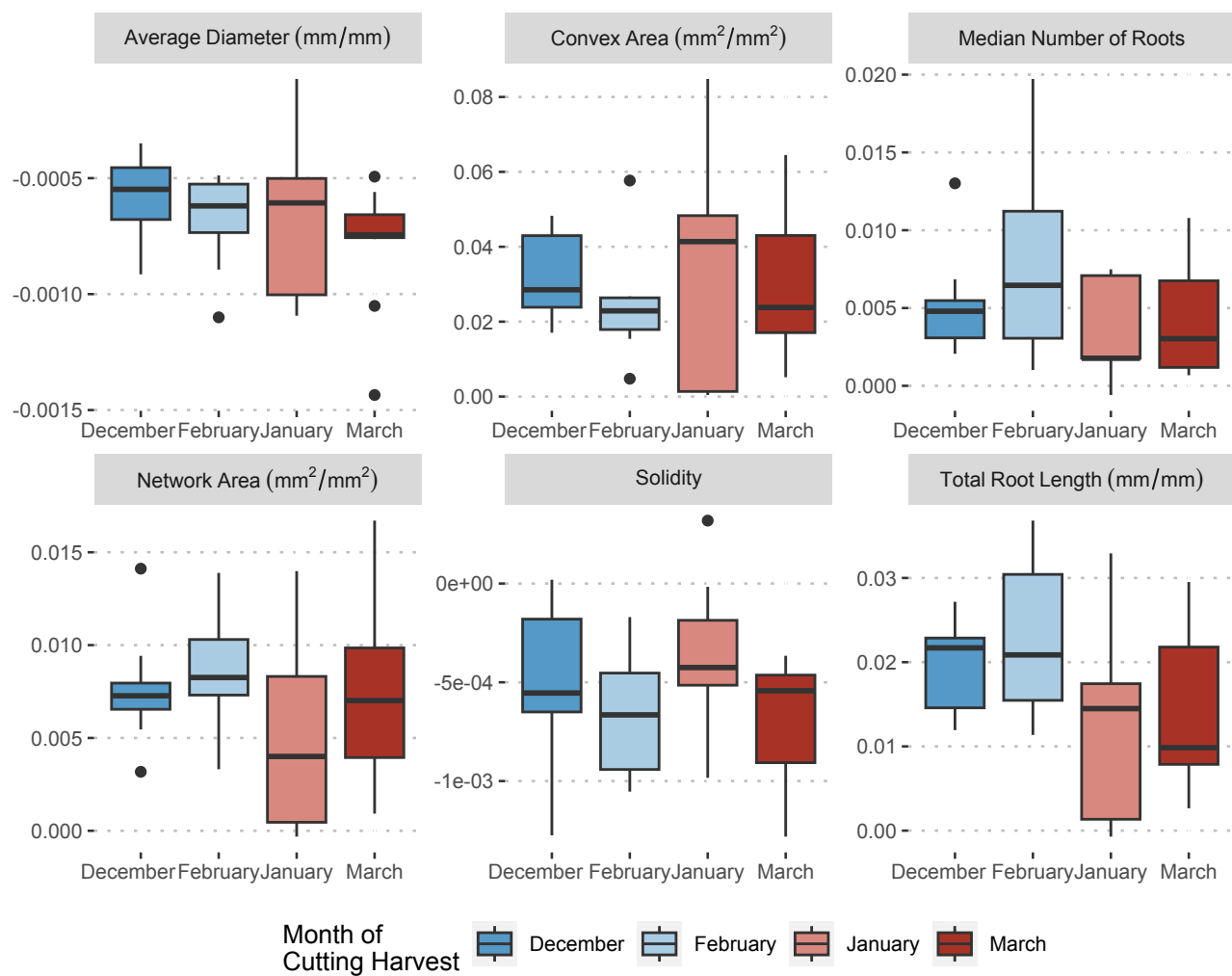

Supplement: Supplementary 1 — Figs. S1 to S7 Tables S1 to S2 [file plantphenomics.0127.f1.zip › Figure_S5.pdf]

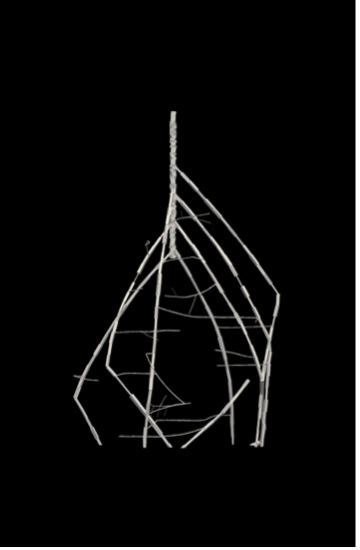

Supplement: Supplementary 1 — Figs. S1 to S7 Tables S1 to S2 [file plantphenomics.0127.f1.zip › Figure_S7a.png]

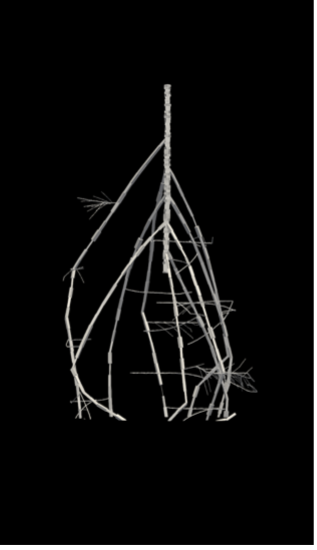

Supplement: Supplementary 1 — Figs. S1 to S7 Tables S1 to S2 [file plantphenomics.0127.f1.zip › Figure_S7b.png]
